# Bmi-1 regulates stem cell-like properties of gastric cancer cells via modulating miRNAs

**DOI:** 10.1186/s13045-016-0323-9

**Published:** 2016-09-20

**Authors:** Xiaofeng Wang, Chang Wang, Xiaowei Zhang, Ruixi Hua, Lu Gan, Mingzhu Huang, Liqin Zhao, Sujie Ni, Weijian Guo

**Affiliations:** 1 Department of Medical Oncology, Fudan University Shanghai Cancer Center, Shanghai, China; 2 Department of Oncology, Shanghai Medical College, Fudan University, Shanghai, China

**Keywords:** Bmi-1, Cancer stem cell, Gastric cancer, miRNAs

## Abstract

**Background:**

B cell-specific Moloney murine leukemia virus integration site 1 (Bmi-1) plays an important role in regulating stemness in some kinds of cancer. However, the mechanisms remain unclear. This study was to investigate whether and how Bmi-1 regulates stemness of gastric cancer.

**Methods:**

We firstly explored the role of Bmi-1 in regulating stem cell-like features in gastric cancer. Secondly, we screened out its downstream miRNAs and clarified whether these miRNAs are involved in the regulation of stemness. Finally, we investigated the mechanisms how Bmi-1 regulates miRNAs.

**Results:**

Bmi-1 positively regulates stem cell-like properties of gastric cancer and upregulates miR-21 and miR-34a. There was a positive correlation between Bmi-1 and miR-21 expression in gastric cancer tissues. MiR-21 mediated the function of Bmi-1 in regulating stem cell-like properties, while miR-34a negatively regulates stem cell-like characteristics via downregulating Bmi-1. Bmi-1 binds to PTEN promoter and directly inhibits PTEN and thereafter activates AKT. Bmi-1 also regulates p53 and PTEN via miR-21. Bmi-1 activated NF-kB via AKT and enhanced the binding of NF-kB to the promoter of miR-21 and miR-34a and increased their expression.

**Conclusions:**

Bmi-1 positively regulates stem cell-like properties via upregulating miR-21, and miR-34a negatively regulates stem cell-like characteristics by negative feedback regulation of Bmi-1 in gastric cancer. Bmi-1 upregulates miR-21 and miR-34a by activating AKT-NF-kB pathway.

**Electronic supplementary material:**

The online version of this article (doi:10.1186/s13045-016-0323-9) contains supplementary material, which is available to authorized users.

## Background

Gastric cancer, with highly aggressivity, is one of common diagnosed and the leading causes of cancer death, so novel biomarker effective therapeutic strategies are being urgently sought [1, 2]. As an oncogene, overexpression of B cell-specific Moloney murine leukemia virus integration site 1 (Bmi-1) is present in a wide variety of tumors and associated with poor prognosis [3, 4]. Bmi-1 may also be involved in cancer metastasis and treatment resistance in some kinds of cancer [5–7]. Our previous studies have found that Bmi-1 expression in gastric cancer is associated with lymph node metastasis and clinical stage and is an independent prognostic factor in patients with gastric cancer [8, 9]. Importantly, Bmi-1 plays an essential role in maintaining self-renewal of hematopoietic stem cells [10, 11]. It is regarded as one of the stem cell markers and a crucial regulator of prostate stem cell self-renewal and malignant transformation [12]. In addition, it is also involved in self-renewal of breast cancer and glioma cancer stem cells (CSCs) [13, 14]. However, the underlying mechanisms of Bmi-1 regulating stemness remain unclear.

CSCs are a small proportion of cells with stem cell features in tumor tissues and considered as a source of tumor formation [15, 16]. Although CSCs only account for a very small fraction of cancer cells, they demonstrate important features including self-renewal, resistance to treatment, and high metastasis potential, all of which make them the source of cancer relapse and treatment failure [17, 18]. Researchers have isolated subgroups of stem cells from many types of tumors. The investigation on gastric CSCs was initiated later than that on other cancers. Scholars have preliminarily isolated gastric CSCs or stem cell-like cells from gastric cancer cell lines or tissues [19–22]. Takaishi et al. conducted serum-free culture of human gastric cancer cell lines to obtain microspheres and found them possessing CSCs properties [19]. CD44^+^CD24^+^ and EpCAM^+^/CD44^+^ cell subgroups have also been confirmed to have CSCs properties [21]. Recently, Chen et al. isolated CD44^+^CD54^+^ cells subgroup from gastric cancer tissue and peripheral blood of patients with gastric cancer and found that this cell subgroup had self-renewal ability and formed transplanted tumor which had features very similar to those of the original tumor [22]. All these studies have shown the existence of gastric CSCs. Side population (SP) cells isolated from gastric cancer cells and gastric cancer tissues also have CSCs features [23, 24]. Bmi-1 was found to be overexpressed in SP cells [24]. However, there are also studies showing no increase of Bmi-1 expression in gastric CSCs [19]. Therefore, the results of Bmi-1 expression in gastric CSCs are still conflicting, and further research is required to clarify whether Bmi-1 plays a role in regulation of stemness in gastric cancer.

In this study, we intended to explore the role and mechanisms of Bmi-1 in regulating stem cell-like features of gastric cancer. Here, we show that Bmi-1 positively regulates stem cell-like properties via upregulating miR-21, and miR-34a negatively regulates stem cell-like characteristics by negative feedback regulation of Bmi-1 in gastric cancer.

## Methods

Details regarding cell strains and cell culture methods, vectors construction and virus infection, spheroid colony formation assay, chemo-sensitivity experiment, cell migration assay, Western blot, immunohistochemical analyses(IHC), immuno-fluorescence staining, Quantitative real time RT-PCR (QRT-PCR), chromatin immunoprecipitation(ChIP), dual fluorescence report assay, and in vivo tumorigenesis are provided in the supplementary materials and methods (see Additional file 1).

### Clinical samples and analyses

The expression of Bmi-1 in 101 paraffin-embedded primary site specimens of gastric cancer and 72 ovarian metastatic specimens originated from gastric cancer, and the expression of Oct4, Sox2, Gli1, CD44, and CD133 in 101 primary site specimens of gastric cancer was tested using IHC.

Another 74 fresh gastric cancer tissues and paired normal mucosal tissues were used to detect the expression of Bmi-1 messenger RNA (mRNA) and miRNAs by QRT-PCR. Details are provided in supplementary methods.

### Statistics

All statistical analyses were done by using the SPSS 19.0 software package, and two-tailed *P* values of less than 0.05 were considered significant. In IHC assays of gastric cancer samples, Pearson *χ*^2^ test was used to determine the correlation between Bmi-1 expression and clinicopathologic characteristics, and Spearman's Rank correlation assay was used to determine the correlation between Bmi-1 and stem cell markers expression. Among 21 pairs of samples, the matching McNemar test was used to detect the difference of Bmi-1 expression between primary and metastatic lesions. In QRT-PCR analysis of fresh tissues, the expression of Bmi-1, miR-21, or miR-34a was not normally distributed. Hence, the distribution was established by using Log10 and geometric averages. The correlation between Bmi-1 and miR-21/miR-34a expression levels was analyzed by the Pearson coefficient test. The correlation between Bmi-1, miR-21, or miR-34a expression and clinicopathologic characteristics was analyzed by ANOVA.

## Results

### Bmi-1 positively regulates stem cell-like properties of gastric cancer cells

**Fig. 1:**
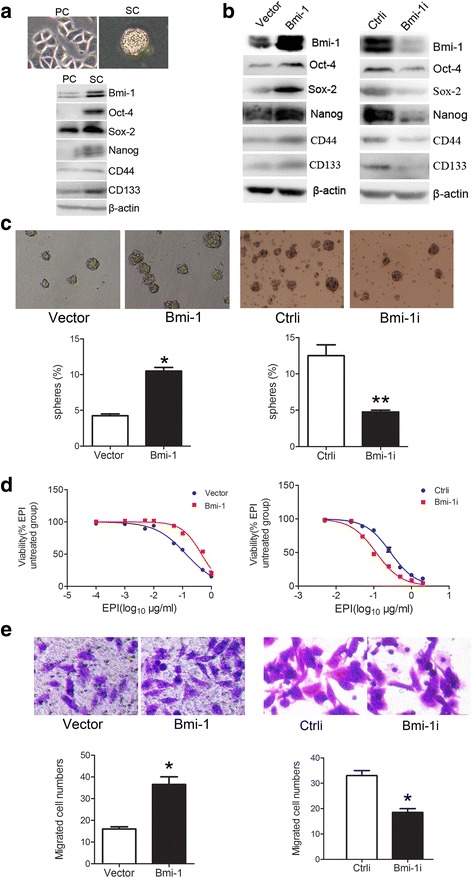
Bmi-1 positively regulates stem cell-like properties of gastric cancer cells. **a** Tumorigenic spheres are derived from SGC7901 gastric cancer cell line in serum-free media containing EGF and bFGF (*upper panel*) and overexpressed stem cell markers including Bmi-1, Oct-4, Sox-2, Nanog, CD44, and CD133 (*lower panel*). The expression of stem cell markers in the cell lysis was analyzed by Western blot. *PC* parental cells, *SC* spheroid cells. **b** Bmi-1 overexpression upregulates the expression of stem cell markers Oct-4, Sox-2, Nanog, CD44, and CD133 in SGC7901 cells (*left panel*) and Bmi-1 knockdown downregulates the expression of stem cell markers in MNK45 cells (*right panel*). The expression of Bmi-1 and stem cell markers in the cell lysis was analyzed by Western blot. **c** Bmi-1 overexpression improves microsphere formation in SGC7901 cells (*left panel*), and Bmi-1 knockdown inhibits microsphere formation in MNK45 cells (*right panel*). Microsphere formation was tested by serum-free suspension culture (*left upper panel*) and quantified (*left lower panel*) in Bmi-1 overexpressing cells (Bmi-1) and control cells (vector) and picture of microsphere (*right upper panel*) and quantified (*right lower panel*) in Bmi-1 knockdown cells (Bmi-1i) and control cells (Ctrli). **d** Bmi-1 overexpression increases drug resistance in SGC7901 cells (IC50: 0.45 μg/ml in overexpression group vs. 0.13 μg/ml in control group, *left panel*) and Bmi-1 knockdown decreases drug resistance to EPI in MNK45 cells (IC50: 0.11 μg/ml in siRNA group vs. 0.28 μg/ml in control group, *right panel*). Cell viability after treatment with increasing doses of EPI for 48 h was determinated with CCK-8. **e** Bmi-1 overexpression promotes migration potential in SGC7901 cells (*left panel*), and Bmi-1 knockdown inhibits migration ability in MNK45 cells (*right panel*). Migration ability of cells was detected by Transwell Assay and then photographed (*upper pane*) and quantified (*lower panel*). *Error bars* in all panels represent the mean ± standard deviation (SD). (*P < 0.05, **P < 0.01 as compared with control)

Cultured CSCs are believed to be able to form spheres that have stem cells properties, which are very similar to endogenous CSCs isolated from human tumor tissues [25, 26]. Our former research has revealed that isolated sphere cells from gastric cancer cell lines and primary cancer cells by serum-free culture method have stem cell-like properties, suggesting microsphere enrich CSCs or stem cell-like cells [27]. To clarify the role of Bmi-1 in stemness of gastric cancer, we checked the expression of Bmi-1 in microsphere and found that sphere cells from SGC7901 and MKN45 cell lines overexpressed Bmi-1 and other stem cell markers Oct-4, Sox2, Nanog, CD44, and CD133 (Fig. 1a and Additional file 2: Figure S1). Next, we used Bmi-1 overexpression plasmids and Bmi-1 interference plasmids to transfect SGC7901 and MKN45 cells, respectively, so as to exogenously change the Bmi-1 expression level. Serum-free suspension culture method was used to detect microsphere formation rate of gastric cancer cells after changing Bmi-1 expression. CCK-8 method was used to detect the influence of Bmi-1 on chemotherapy sensitivity of gastric cancer cells, and Transwell Chambers as an in vitro migration model were used to detect the effects of Bmi-1 on the migration ability of gastric cancer cells. Results showed that the microsphere formation rate was significantly higher, the drug resistance to epirubicin (EPI) was increased by about three times (IC50: 0.45 vs. 0.13 μg/ml), and the cells migration ability was enhanced after overexpressing Bmi-1 in SCG 7901 cells, when compared with those in control cells (left panels of Fig. 1c–e). For MKN45 cells, by contrast, the microsphere formation rate was decreased, chemotherapy sensitivity to EPI was increased (IC50: 0.11 μg/ml in small interfering RNA (siRNA) group vs. 0.28 μg/ml in control group), and cell migration ability was suppressed after Bmi-1 interference (right panels of Fig. 1c–e). We have also tested the influence of Bmi-1 on stem cell markers by Western blot and found that Bmi-1 upregulated the stem cell markers including CD44, CD133, Nanog, SOX2, and Oct-4 (Fig. 1b). In another gastric cancer cell line AGS, we decreased Bmi-1 expression by gene interference or overexpressed Bmi-1 and obtained similar results by the same methods (data not shown). These results showed that Bmi-1 positively regulates stem cell-like characteristics of gastric cancer cells.

In order to further verify the relationship between Bmi-1 and stem cell-like characteristics, we used immunohistochemical method to detect the expression of Bmi-1 in 101 primary site specimens of gastric cancer and 72 ovarian metastases specimens originated from gastric cancer (Additional file 3: Figure S2a). The results showed that 63 out of 101 primary site specimens were Bmi-1 protein positive with a positive rate of 62.4 %, and Bmi-1 expression positively correlated with regional lymph node metastasis (Table 1). Among 72 ovarian metastases specimens, 57 were Bmi-1 protein positive with a positive rate of 79.2 %, which was significantly higher than that in primary tumors (*P* = 0.018). Among 21 pairs of samples for which the primary tumor and ovarian metastases were from the same patient, 6 pairs had inconsistent expression of Bmi-1, including 5 pairs with the ovarian metastasis showing positive Bmi-1 and the primary tumor showing negative Bmi-1, 1 pair with the ovarian metastasis showing negative Bmi-1 and the primary tumor showing positive Bmi-1, and the matching McNemar test demonstrated a trend of higher expression in the metastatic site than in the primary site (*P* = 0.063, Additional file 4: Table S1). We also detected the expression of stem cell markers including Oct-4, Sox-2, Glil, CD44, and CD133 in the primary sites of gastric cancer and analyzed the correlation between these stem cell markers and Bmi-1 by spearman rank correlation test (Additional file 3: Figure S2b–f, and Additional file 5: Table S2). The results showed that Bmi-1 expression was positively correlated with the expression of the above-mentioned stem cell markers.

**Table 1:** The relation between the expression of Bmi-1 protein and clinicopathologic variables

Variables	Bmi-1(+)	Bmi-1(−)	*P* value
Lymph node metastasis			
Negative	7(33.3 %)	14(66.7 %)	0.003
Positive	51(68.9 %)	23(31.1 %)	
T classification			
T1–3	22(57.9 %)	16(42.1 %)	0.606
T4	36(63.2 %)	21(36.8 %)	
TNM staging			
I	2(50 %)	2(50 %)	0.254
II	10(45.5 %)	12(54.5 %)	
III	38(69.1 %)	17(30.9 %)	
IV	8(57.1 %)	6(42.9 %)	
Differentiation			
Moderately	25(59.5 %)	17(40.5 %)	0.786
Poorly	33(62.3 %)	20(37.7 %)	
Vascular invasion			
Positive	39(65.0 %)	21(35.0 %)	0.302
Negative	19(54.3 %)	16(45.7 %)	
Neural invasion			
Positive	35(61.4 %)	22(38.6 %)	0.932
Negative	23(60.5 %)	15(39.5 %)	
Age(year)			
≥60	22(57.9 %)	16(42.1 %)	0.606
<60	36(63.2 %)	21(36.8 %)	
Gender			
Male	41(61.2 %)	26(38.8 %)	0.965
Female	17(60.7 %)	11(39.3 %)	

### Bmi-1 regulates the expression of several miRNAs

**Fig. 2:**
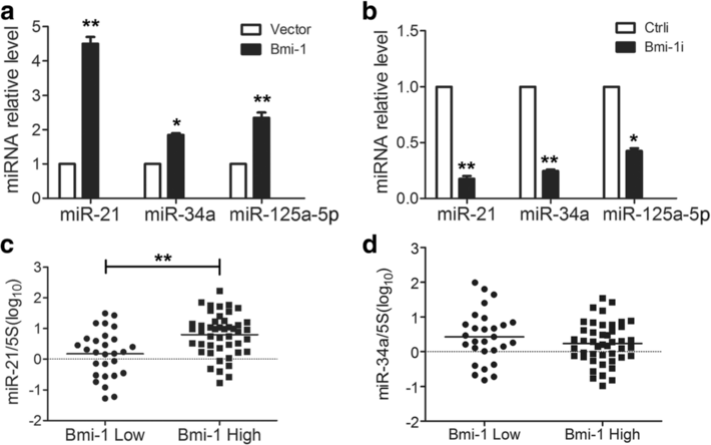
Bmi-1 regulates the expression of several miRNAs. **a** Bmi-1 overexpression upregulates the expression of miR-21, miR-34a, and miR-125a-5p. Fold change of miRNAs was detected by QRT-PCR in SGC7901 cells with Bmi-1 overexpression and control cells. **b** Bmi-1 knockdown downregulates the expression of miR-21, miR-34a, and miR-125a-5p. Fold change of miRNAs was detected by QRT-PCR, normalized to 5S in MKN45 cells with Bmi-1 knockdown and control cells. **c** Pearson correlation analysis showed there was a positive correlation between the expression level of Bmi-1 and miR-21. The expression of miR-21 in gastric cancer tissues with high Bmi-1 expression was higher than tissues with low Bmi-1 expression. The log10 of miR-21 expression level was plotted in Bmi-1 high and Bmi-1 low groups of gastric cancer tissues. **d** The expression of miR-34a was not different in gastric cancer tissues with high Bmi-1 expression from tissues with low Bmi-1 expression. The log10 of miR-34a expression level was plotted in Bmi-1 high and Bmi-1 low groups of gastric cancer tissues. *Error bars* in all panels represent the mean ± SD (*P < 0.05, **P < 0.01)

Previous study has shown that the transcription factor p53 regulates epithelial-mesenchymal transition (EMT) and stem cell properties through modulating miRNAs [28]. In light of this, we assumed that as an exogenous gene-silencing factor which regulates the expression of a variety of genes [29–31], Bmi-1 may also play its biological effects via regulating the expression of miRNAs. To test this hypothesis, we used microRNA (miRNA) expression profile chip to detect the changes of miRNA in gastric cancer cells after Bmi-1 knockdown. The results showed that there were eight differentially expressed miRNAs, in which miR-21, miR-210, miR-886-5p, miR-103, miR-107, and miR-34a were positively regulated by the Bmi-1, while miR-15b and miR-125-a-5p were negatively regulated by Bmi-1. Validating through QRT-PCR, we found three miRNAs with significantly altered expression: miR-21, miR-34a, and miR-125-a-5p, and their expression were all positively regulated by Bmi-1 (Fig. 2a, b).

In order to further verify the relationship between Bmi-1 and miRNAs, we used QRT-PCR method to detect Bmi-1 mRNA and miR-21, miR-34a levels in the gastric cancer tissues, and corresponding normal gastric mucosa tissues from 74 patients with gastric cancer. Pearson correlation analysis showed that there was a positive correlation between the expression of Bmi-1 and miR-21 (*r* = 0.260, *P* = 0.025) as the expression of miR-21 in gastric cancer tissues with high Bmi-1 expression was higher than that in tissues with low Bmi-1 expression (Fig. 2c), while the expression of miR-34a had no obvious correlation with Bmi-1 level (*r* = −0.118, *P* = 0.318) as the expression of miR-34a was not different in gastric cancer tissues with high Bmi-1 expression from tissues with low Bmi-1 expression (Fig. 2d). We also analyzed the correlation between the Bmi-1, miR-21, and clinical pathological factors. Results showed that the expression of Bmi-1 in cancer tissues is upregulated in 37 cases (50 %) compared with that in paired adjacent normal tissues (Additional file 6: Table S3), and Bmi-1 overexpression positively correlated with depth of invasion (T classification), vascular invasion, or neural invasion as the expression of Bmi-1 was higher in patients with deeper invasion, positive vascular invasion, or neural invasion (Table 2), which suggested that overexpression of Bmi-1 correlated with a more aggressive phenotype in GC. On the other hand, the expression of miR-21 in cancer tissues is upregulated in 44 cases (59.46 %) compared with that in paired adjacent normal tissues (Additional file 6: Table S3), and the expression of miR-21 was associated with vascular invasion or differentiation as the expression of miR-21 was higher in patients with vascular invasion or poor differentiation (Table 2), which suggested that overexpression of miR-21 correlated with a more aggressive phenotype. These data also suggested miR-21 might be a downstream effector of Bmi-1.

**Table 2:** Correlations between the expression level of Bmi-1, miR-21 or miR-34a, and clinical-pathologic variables

Variable	Number	Bmi-1		miR-21		miR-34a	
		GA	*P*	GA	*P*	GA	*P*
Age(year)							
≤59	40	2.246		2.066		0.449	
≥60	34	2.699	0.616	2.596	0.681	0.747	0.204
Gender							
Male	56	2.373		1.984		0.434	
Female	18	2.682	0.774	3.608	0.351	0.619	0.449
Lymph node metastasis							
Negative	30	1.717		1.988		0.679	
Positive	44	3.110	0.108	2.531	0.667	0.502	0.459
T classification							
T1/2/3	36	1.372		1.542		0.733	
T4	38	4.223	0.001*	3.343	0.159	0.445	0.212
Vascular invasion							
Negative	33	1.235		1.080		0.892	
Positive	41	4.235	0.000*	4.210	0.012*	0.394	0.041*
Neural invasion							
Negative	31	1.348		1.654		0.833	
Positive	43	3.755	0.004*	2.905	0.313	0.430	0.101
Differentiation							
Moderately	32	1.687		1.013		0.612	
Poorly	42	3.243	0.074	4.278	0.008*	0.536	0.744
Staging							
I/II	27	1.935		1.442		0.818	
III/IV	47	2.795	0.332	2.997	0.200	0.460	0.165

*GA* geometrical average

*Statistically significant

### MiR-21 positively regulates stem cell-like characteristics of gastric cancer cells

We intended to clarify whether Bmi-1 downstream miRNAs is involved in the regulation of stemness in gastric cancer cells. First of all, we investigated miR-21 which is closely related to Bmi-1.

**Fig. 3:**
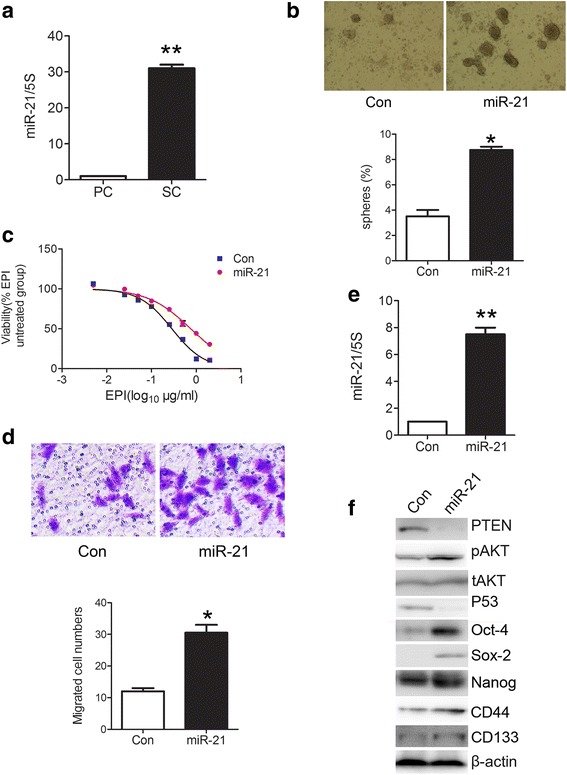
miR-21 overexpression enhances stem cell-like properties of gastric cancer cells. **a** miR-21 is overexpressed in cancer stem-like cells of gastric cancer. Fold change of miR-21 in spheroid cells (SC) and parental cells (PC) of SGC7901 was analyzed by QRT-PCR. **b** miR-21 overexpression increases microsphere formation rate in gastric cancer cells. Microsphere formation rate was detected by serum-free culture (*upper panel*) and quantified (*lower panel*) in miR-21 overexpressing cells (miR-21) and control cells (Con). **c** miR-21 overexpression increases drug resistance in SGC7901 cells. Cell viability in miR-21 overexpressing cells (miR-21) and control cells (Con) treated with different doses of EPI for 48 h was determinated with CCK-8. **d** miR-21 overexpression promotes migration potential in SGC7901 cells. Migration ability of cells was detected by Transwell Assay and then photographed (*upper pane*) and quantified (*lower panel*). **e** miR-21 overexpression was confirmed in SGC7901 cells after infected by lentivirus with miR-21 overexpression. Fold change of miR-21 in miR-21 overexpressing cells (miR-21) and control cells (Con) was analyzed by QRT-PCR. **f** miR-21 overexpression upregulates the expression of stem cell markers in SGC7901 cells. The expression of stem cell markers and known miR-21 target and downstream genes (PTEN-AKT, P53) in the cell lysis was analyzed by Western blot. *Error bars* in all panels represent the mean ± SD (*P < 0.05, **P < 0.01 as compared with control)

At first, we used QRT-PCR to detect the expression of miR-21 in suspension microspheres separated from gastric cancer cells by serum-free culture method. The results showed that miR-21 expression in suspension microspheres which enrich stem-like cells increased significantly than in the parent adherent cells (Fig. 3a). Furthermore, we tested the influence of different miR-21 expression levels on stem cell-like characteristics and found that miR-21 upregulation can increase the microsphere formation rate, resistance to chemotherapy, and migration ability of gastric cancer cells (Fig. 3b-d), while miR-21 downregulation can decrease the microsphere formation rate, resistance to chemotherapy, and migration ability (Additional file 7: Figure S3a–c). We also tested the effect of miR-21 on the expression of stem cell markers and found that the expression of CD44, CD133, Nanog, SOX2, and Oct-4 were increased after miR-21 overexpression in SGC7901 cells (Fig. 3f) and reduced after miR-21 downregulation in MKN45 cells (Additional file 7: Figure S3e). These results indicated that miR-21 may positively regulate the stem cell-like characteristics of gastric cancer cells.

Two important tumor suppressor genes PTEN and p53 are downstream target genes of miR-21 [32, 33]. We also tested the effect of miR-21 on these two genes. The results demonstrated that after overexpression of miR-21 in SGC7901 cells, the expression of PTEN, and P53 was decreased and the expression of phosphorylated AKT (pAKT) of the PTEN downstream was increased and total AKT had no change (Fig. 3f); after downregulation of miR-21 in MKN45 cells, PTEN, and P53 were increased, and pAKT was downregulated (Additional file 7: Figure S3e).

### Bmi-1 regulates stem cell-like characteristics of gastric cancer cells via upregulation of miR-21

**Fig. 4:**
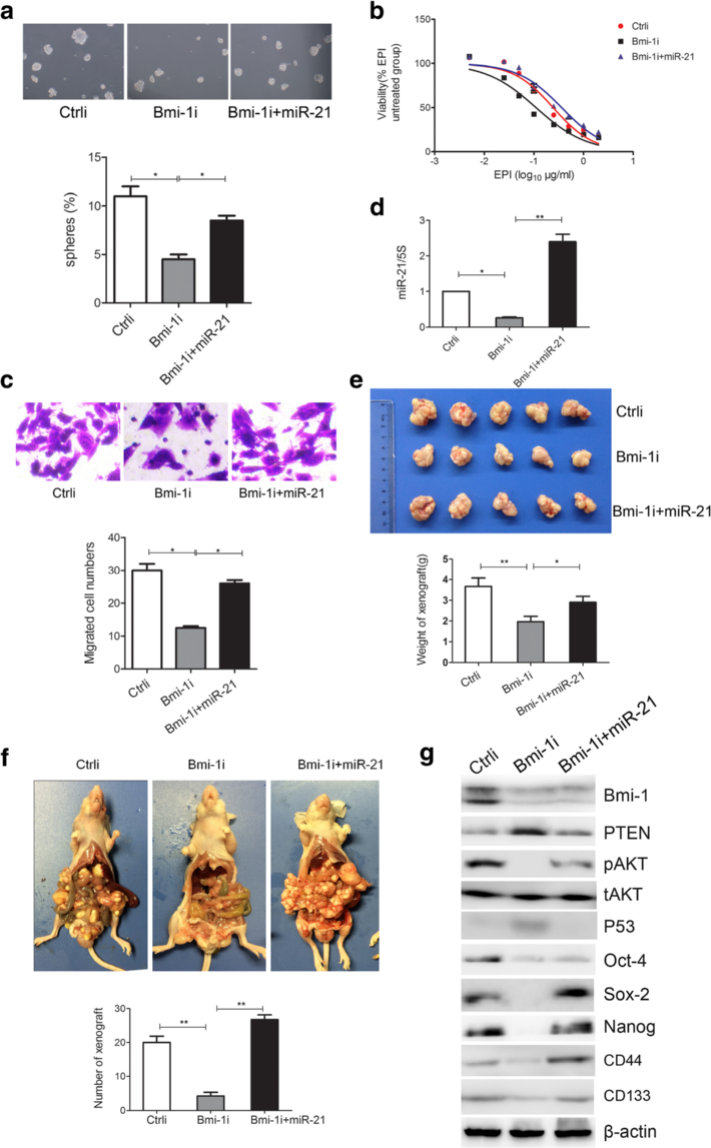
miR-21 overexpression restores stem cell-like characteristics of gastric cancer cells which were inhibited by Bmi-1 knockdown. **a** miR-21 overexpression restores microsphere formation of Bmi-1 knockdown MKN45 cells. Microsphere formation in control cells (Ctrli), Bmi-1 gene interference cells (Bmi-1i), and cells with Bmi-1 interference and simultaneous overexpression of miR-21 (Bmi-1i+miR-21) was detected by serum-free suspension culture (*upper panel*) and quantified (*lower panel*). **b** miR-21 overexpression increases drug resistance in MKN45 cells which was inhibited by Bmi-1 knockdown. Cell viability in Ctrli, Bmi-1i, and Bmi-1i+miR-21 MKN45 cells treated with different doses of EPI for 48 h was determinated with CCK-8 method. **c** miR-21 overexpression enhances migration ability in MKN45 cells which was inhibited by Bmi-1 knockdown. Migration ability in Ctrli, Bmi-1i, and Bmi-1i+miR-21 MKN45 cells was tested by Transwell migration assay. **d** miR-21 overexpression in Bmi-1 knockdown MKN45 cells was confirmed by QRT-PCR. Fold change of miR-21 in Ctrli, Bmi-1i, and Bmi-1i+miR-21 MKN45 cells was determinated by QRT-PCR. **e** miR-21 overexpression enhances tumorigenecity in MKN45 cells which was inhibited by Bmi-1 knockdown. In vivo tumorigenecity in Ctrli, Bmi-1i, and Bmi-1i+miR-21 MKN45 cells was detected by subcutaneously cancer cells injected SCID mice model. **f** miR-21 overexpression enhances implantation metastasis of MKN45 cells which was inhibited by Bmi-1 knockdown. In vivo implantation metastasis potential in Ctrli, Bmi-1i, and Bmi-1i+miR-21 MKN45 cells was detected by intraperitoneal cancer cells injected SCID mice model. **g** miR-21 overexpression restores the expression of stem cell markers and miR-21 target genes (PTEN, P53) in Bmi-1 knockdown MKN45 cells. The expression of Bmi-1, stem cell markers and miR-21 target genes (PTEN-AKT, P53) in Ctrli, Bmi-1i and Bmi-1i+miR-21 MKN45 cells was analyzed by Western blot. *Error bars* in all panels represent the mean ± SD (*P < 0.05, **P < 0.01)

In order to detect whether miR-21 plays a role in the downstream of Bmi-1, we utilized co-transfection to simultaneously change the expression of Bmi-1 and miR-21 in cells in an exogenous way and observed the effects on stem cell-like properties of gastric cancer cells. In MKN45 cells, we compared the stem cell-like characteristics of the control cells, Bmi-1 gene interference cells and cells with Bmi-1 interference, and simultaneous overexpression of miR-21. The results showed that miR-21 overexpression can reverse the inhibition of stem cell-like characteristics (self-renewal ability, resistance to chemotherapy and cell migration ability) (Fig. 4a–c) and the downregulation of stem cell markers expression caused by Bmi-1 knockdown (Fig. 4g). Further, miR-21 overexpression enhances in vivo tumorigenecity and implantation metastasis of MKN45 cells which was inhibited by Bmi-1 knockdown in SCID mice model (Fig. 4e, f). In SGC7901 cells, downregulation of miR-21 can inhibit the enhancement of stem cell-like characteristics and the increase of stem cell markers expression caused by Bmi-1 overexpression (Additional file 8: Figure S4a–c, e). These results indicated Bmi-1 can regulate stem cell-like characteristics of gastric cancer cells by upregulation of miR-21.

We further tested the change of miR-21 downstream target genes and found that Bmi-1 overexpression can downregulate p53 and PTEN, which are downstream target genes of miR-21, and upregualte pAKT, while simultaneous downregulation of miR-21 can restore the expression of p53 and PTEN and downregulate pAKT (Additional file 8: Figure S4e). Similarly, when Bmi-1 is silenced, p53 and PTEN expression are upregulated, and pAKT is downregulated, while overexpression of miR-21 at the same time can inhibit the upregulation of p53 and PTEN and the downregulation of pAKT caused by Bmi-1 silence (Fig. 4g). These results further confirm that Bmi-1 regulates the expression of miR-21 and its downstream target genes and pathways.

### MiR-34a negatively regulates stem cell-like characteristics of gastric cancer cells

**Fig. 5:**
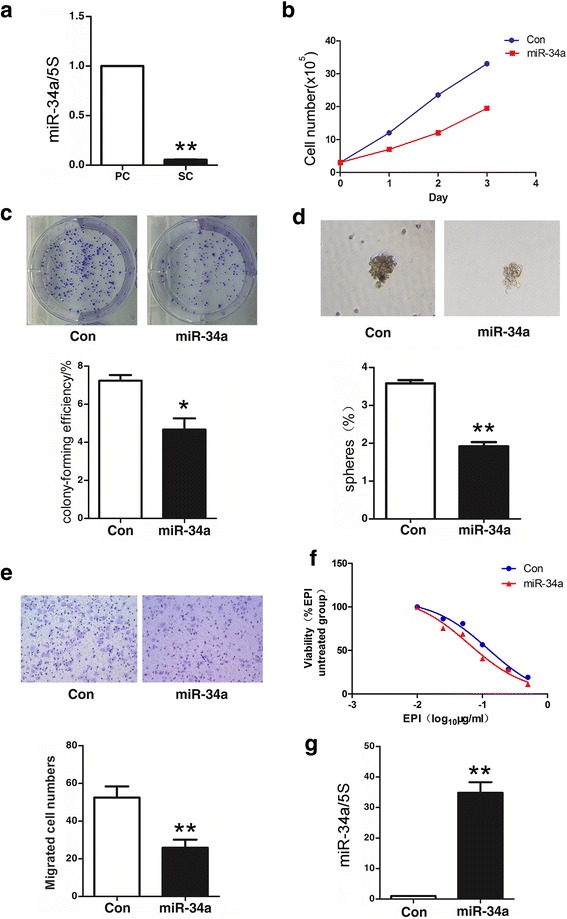
miR-34a negatively regulates stem cell-like properties of gastric cancer cells. **a** Expression of miR-34a was decreased in microsphere of MKN45 cells. Fold change of miR-34a in spheroid cells (SC) and parental cells (PC) was analyzed by QRT-PCR. **b** miR-34a overexpression inhibits cell growth in MNK45 cells. Cells growth was detected by CCK-8 in miR-34a overexpressing cells (miR-34a) and control cells (Con). **c** miR-34a overexpression inhibits proliferation in MNK45 cells. Proliferation ability of cells was detected by colony formation assay, then photographed (*upper panel*) and quantified (*lower panel*). **d** miR-34a overexpression decreases microsphere formation in MKN45 cells. Microsphere formation was tested by serum-free suspension culture (*upper panel*) and quantified (*lower panel*) in miR-34a overexpressing cells (miR-34a) and control cells (Con). **e** miR-34a overexpression inhibits migration ability in MNK45 cells. Migration ability of cells was detected by Transwell Assay and then photographed (*upper panel*) and quantified (*lower panel*). **f** miR-34a overexpression decreases drug resistance to EPI in MKN45 cells. Cell viability after treatment with increasing doses of drug for 48 h was determined with CCK-8. **g** miR-34a overexpression in MKN45 cells after infecting by lentivirus containing miR-34a was confirmed by QRT-PCR. *Error bars* in all panels represent the mean ± SD (*P < 0.05, **P < 0.01 as compared with control)

MiR-34a plays a role of tumor suppressor in other types of tumors [34, 35]. As we found Bmi-1 upregulates miR-34a in gastric cancer cells, here, we explored the function of miR-34a in gastric cancer. Firstly, we found the expression of miR-34a was downregulated in all six gastric cancer cell lines compared with that in human gastric mucosal epithelial cell line GES-1 (Additional file 9: Figure S5a), and it was also downregulated in suspension microspheres than in the parent adherent cells (Fig. 5a). Overexpression of miR-34a inhibited cells growth and colony formation (Fig. 5b, c), suggesting it may act as a tumor suppressor. Further, we examined the effect of different expression levels of miR-34a on stem cell-like characteristics and found that upregulation of miR-34a may suppress the formation rate of the microspheres of gastric cancer cells, resistance to chemotherapy and cell migration (Fig. 5d–f), while downregulation of miR-34a can improve the formation of microspheres, chemotherapy resistance, and cell migration (Additional file 9: Figure S5b–d). Further, we analyzed the expression of miR-34a in 74 patients with gastric cancer. Results showed that the expression of miR-34a in cancer tissues is downregulated in 35 cases (47.3 %) compared with that in paired adjacent normal tissues (Additional file 6: Table S3), and miR-34a expression negatively correlated with vascular invasion as the expression of miR-34a was lower in patients with positive vascular invasion (Table 2), which suggested that downregulation of miR-34a correlated with a more aggressive phenotype. These results suggest that miR-34a might act as a tumor suppressor and negatively regulates stem cell-like characteristics in gastric cancer.

### MiR-34a negatively regulates stem cell-like characteristics of gastric cancer cells by downregulating Bmi-1

**Fig. 6:**
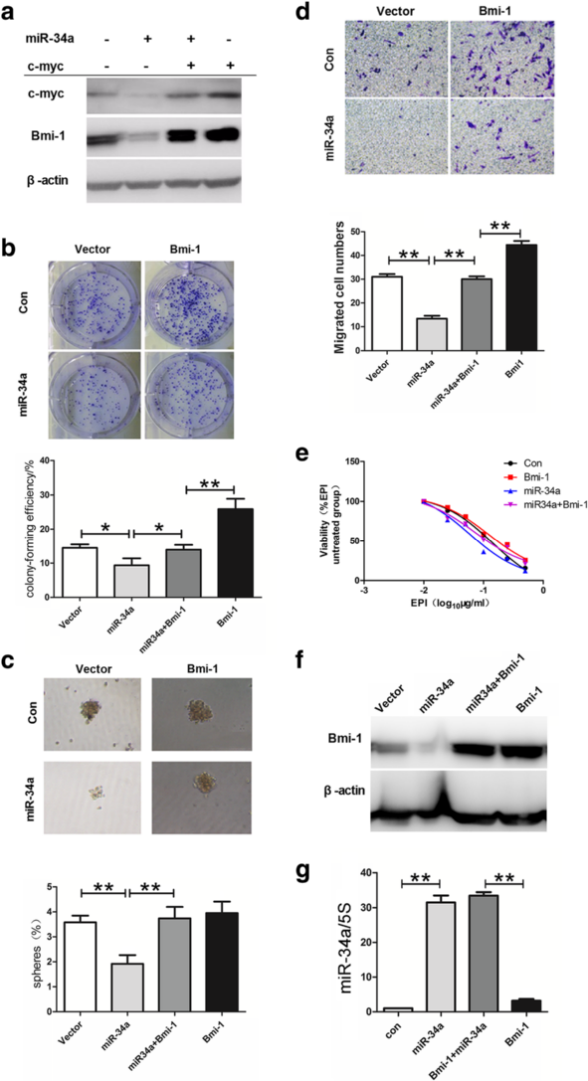
MiR-34a negatively regulates stem cell-like characteristics of gastric cancer cells by downregulating Bmi-1. **a** Expression of Bmi-1 and c-Myc was suppressed by miR-34a, while overexpression of c-Myc increased the inhibited expression of Bmi-1. The expression of c-Myc, Bmi-1, and β-actin in the cell lysis was analyzed by Western blot. **b** Overexpression of Bmi-1 reversed the inhibition of proliferation ability(colony formation) induced by miR-34a upregulation in MKN45 cells and then photographed (*upper panel*) and quantified (*lower panel*). **c** Overexpression of Bmi-1 reversed the inhibition of microsphere formation induced by miR-34a upregulation. Microsphere formation was tested by serum-free suspension culture (*upper panel*) and quantified (*lower panel*) in control cells (vector), miR-34a overexpressing cells(miR-34a), cells simultaneously overexpressing Bmi-1 and miR-34a (miR34a+Bmi-1), and Bmi-1 overexpressing cells (Bmi-1). **d** Overexpression of Bmi-1 reversed the inhibition of migration ability induced by miR-34a upregulation in MKN45 cells. Migration ability of cells was detected by Transwell Assay and then photographed (*upper panel*) and quantified (*lower panel*). **e** Overexpression of Bmi-1 reversed the inhibition of drug resistance induced by miR-34a upregulation in MKN45 cells. Cell viability after treatment with increasing doses of drug EPI for 48 h was determined with CCK-8. **f** and **g** Bmi-1 overexpression in MKN45 cells was confirmed by Western blot (*f*) and miR-34a overexpression in MKN45 cells after infecting by lentivirus containing miR-34a was confirmed by QRT-PCR (*g*) in control cells (con), miR-34a overexpressing cells (miR-34a), cells simultaneously overexpressing Bmi-1 and miR-34a (miR34a+Bmi-1) and Bmi-1 overexpressing cells (Bmi-1) . *Error bars* in all panels represent the mean ± SD (*P < 0.05, **P < 0.01)

In the above-mentioned study, we found that Bmi-1 can upregulate the expression of miR-34a, but the function of Bmi-1 in regulating the stem cell-like characteristics is in contrary to that of miR-34a. How to explain this phenomenon? We assumed there might be a negative feedback pathway and Bmi-1 may inhibit its own function by this pathway. We used several software to predict miR-34a target gene. Bmi-1 was not among the predicted target genes, but Myc appeared to be its target gene. Previous research also showed that c-Myc was a target gene of miR-34 [36, 37]. We previously found that c-Myc can bind to Bmi-1 promoter and increase the expression of Bmi-1 [38]. Therefore, miR-34a may theoretically downregualte Bmi-1 via c-Myc. We examined the effect of miR-34a on c-Myc and Bmi-1 and found that after upregulation of miR-34a, the expression of both Bmi-1 and c-Myc was suppressed, while overexpression of c-Myc may increase the inhibited expression of Bmi-1, suggesting that miR-34a may downregulate Bmi-1 through targeting c-Myc (Fig. 6a).

To determine whether miR-34a plays a role via Bmi-1, we used the co-transfection method to simultaneously change the expression of Bmi-1 and miR-34a in an exogenous way and observe the effect on characteristics of gastric cancer stem cells. In AGS and MKN45 cells, we compared stem cell-like characteristics of the control cells, miR-34a overexpressing cells, and cells simultaneously overexpressing Bmi-1 and miR-34a and found that overexpression of Bmi-1 can reverse the inhibition of stem cell-like characteristics (colony and microsphere formation, cell migration and resistance to chemotherapy) induced by miR-34a upregulation (Fig. 6b–e). These results suggested that miR-34a negatively regulates stem cell-like characteristics of gastric cancer cells by negative feedback regulation of Bmi-1.

### Bmi-1 regulates the expression of miR-21 and miR-34a via NF-kB

**Fig. 7:**
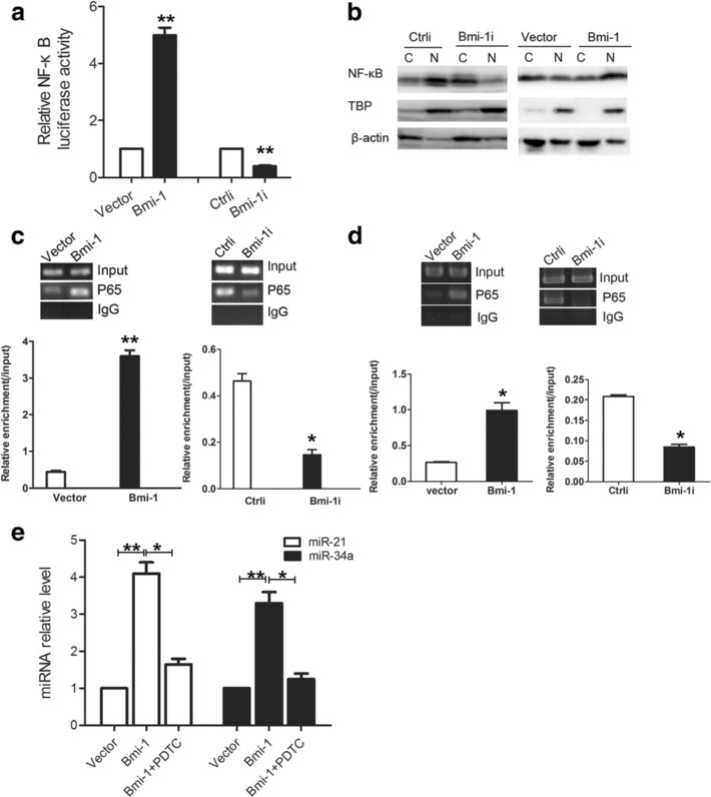
Bmi-1 regulates the expression of miR-21 and miR-34a via NF-kB. **a** Bmi-1 overexpression increases NF-kB transcriptional activity in SGC7901 cells, and Bmi-1 knockdown reduces NF-kB transcriptional activity in MKN45 cells. Gastric cancer cells were co-transfected with pNF-kB-luciferase plasmid and pRL-TK renilla plasmid, and transcriptional activity of NF-kB was tested by luciferase reporter activity assays. **b** Bmi-1 overexpression induces NF-kB aggregation in nuclear of SGC7901 cells, and Bmi-1 knockdown decreases NF-kB aggregation in nuclear of MKN45 cells. Cytoplasmic(C) and nuclear (N) proteins of cells were extracted and detected by Western blot. Nuclear protein TBP was used as a nuclear protein internal control and β-actin was as a loading control. **c** Bmi-1 overexpression promotes the binding of p65 to miR-21 promoter region in SGC7901 cells, and Bmi-1 interference reduces the binding of p65 to miR-21 promoter region in MKN45 cells. The binding of p65 to miR-21 promoter was analyzed by ChIP in gastric cancer cells with changed Bmi-1 expression level. The ChIP-enriched DNA analyzed by QRT-PCR was normalized to input DNA, followed by subtracting nonspecific binding determined by control IgG. **d** Bmi-1 overexpression promotes the binding of p65 to miR-34a promoter region in SGC7901 cells, and Bmi-1 interference reduces the binding of p65 to miR-34a promoter region in MKN45 cells. The binding of p65 to miR-34a promoter was analyzed by ChIP in gastric cancer cells with changed Bmi-1 expression level. The ChIP-enriched DNA analyzed by QRT-PCR was normalized to input DNA, followed by subtracting nonspecific binding determined by control IgG. **e** NF-kB inhibitor PDTC treatment inhibits the increase of expression of miR-21 and miR-34a induced by Bmi-1 overexpression in SGC7901 gastric cancer cells. Fold changes of miR-21 and miR-34a in control vector transfected cells (vector), Bmi-1 overexpressing cells (Bmi-1), and Bmi-1 overexpressing cells treated with NF-kB inhibitor PDTC (Bmi-1+PDTC) was analyzed by QRT-PCR. *Error bars* in all panels represent the mean ± SD (*P < 0.05, **P < 0.01)

As Bmi-1 is a gene-silencing factor, our results showed that Bmi-1 has a positively regulatory effect on miR-21 and miR-34a, indicating the existence of an intermediate link in such a regulatory effect on miR-21 and miR-34a. Previous studies have shown that Bmi-1 can increase the NF-kB activity [39], and the miR-21 and miR-34a promoter region has the NF-kB binding site [40]. Based on these findings, we assumed that Bmi-1 may regulate the expression of miR-21 and miR-34a via NF-kB. Firstly, we utilized dual luciferase report gene assay to detect the NF-kB transcriptional activity after exogenous change in Bmi-1 expression and found that after upregulation of Bmi-1 in SGC7901 cells, the NF-kB transcription activity was enhanced significantly; and after downregulation of Bmi-1 in MKN45 cells, the NF-kB transcription activity was decreased significantly (Fig. 7a). As the NF-kB activity is related to its aggregation in cell nucleus, we used Western blot to measure the relative content of NF-kB subunit p65 after extraction of the nucleus protein and plasma protein, in order to detect changes in the intracellular distribution of p65. After Bmi-1 overexpression in SGC7901 cells, the p65 level in the nucleus was increased; while after Bmi-1 interference in MKN45 cells, the p65 level in the nuclear was decreased (Fig. 7b). These results confirmed that Bmi-1 can promote the NF-kB aggregation in nuclear and activate its transcription activity in gastric cancer cells. Then, we validated whether the NF-kB may bind to miR-21 and miR-34a promoter by ChIP test. Results showed that p65 do bind to the promoter region of miR-21 and miR-34a, and after Bmi-1 overexpression in SGC7901 cells, the binding of p65 to the miR-21 and miR-34a promoter region was significantly increased (left panel of Fig. 7c, d), while after Bmi-1 interference in MKN45 cells, the binding of p65 to the miR-21 and miR-34a promoter region was decreased significantly (right panel of Fig. 7c, d), indicating that Bmi-1 may increase the binding of p65 to the miR-21 and miR-34a promoter region. Furthermore, we used the NF-kB inhibitor pyrrolidine dithiocarbamate (PDTC) to treat SGC7901 cells overexpressing Bmi-1 and found PDTC treatment can inhibit the increase of miR-21 and miR-34a expression induced by Bmi-1 overexpression (Fig. 7e).These results suggested that Bmi-1 regulates the expression of miR-21 and miR-34a via NF-kB.

### Bmi-1 regulates N F-kB-miR-21/miR-34a via activating AKT

**Fig. 8:**
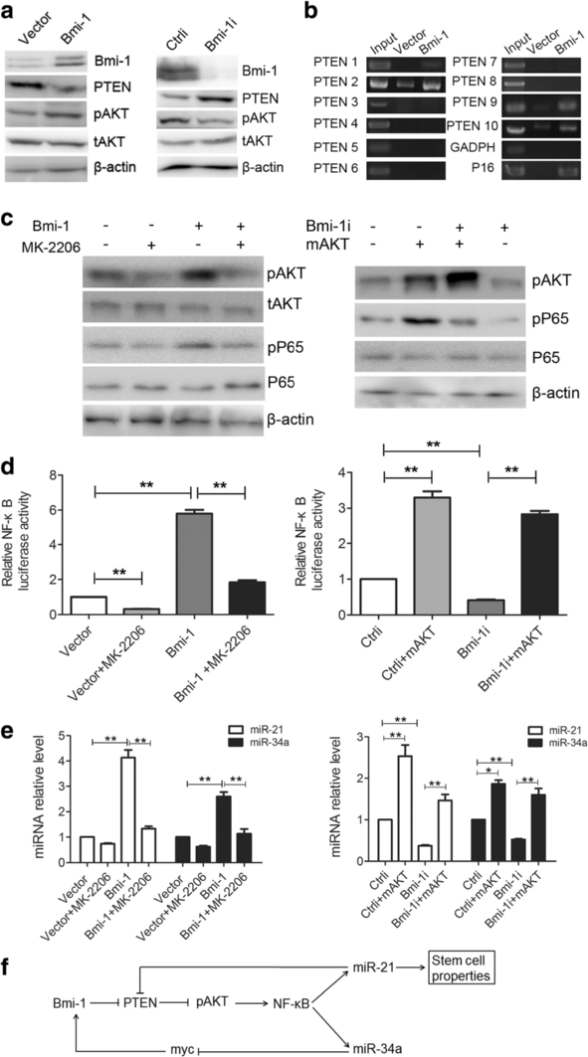
Bmi-1 regulates NF-kB - miR-21/miR-34a via activating AKT. **a** Bmi-1 overexpression downregulates the expression of PTEN and increases the expression of phosphalated AKT (pAKT) in SGC7901 cells (*left panel*), and Bmi-1 knockdown upregulates the expression of PTEN and decreases the expression of pAKT in MKN45 cells (*right panel*). The expression of Bmi-1, PTEN, pAKT, and total AKT (tAKT) was analyzed by Western blot in gastric cancer cells. **b** Bmi-1 overexpression promotes the binding of p65 to the PTEN promoter region in SGC7901 cells. The binding of Bmi-1 to PTEN promoter was detected by ChIP in SGC7901 cells. The p16 promoter was used as positive control and GAPDH as negative control. **c** AKT inhibitor MK-2206 treatment reduces the expression of phosphalated p65 (pp65) induced by Bmi-1 overexpression (*left panel*), and activated AKT can increase the expression of pp65 inhibited by Bmi-1 knock down (*right panel*). The effect of Bmi-1 and AKT on pp65 and total p65 expression was tested by Western blot. **d** AKT inhibitor MK-2206 treatment suppresses NF-kB transcriptional activity induced by Bmi-1 overexpression (*left panel*), and activated AKT promotes NF-kB transcriptional activity inhibited by Bmi-1 knock down (*right panel*). Transcriptional activity of NF-kB in gastric cancer cells was tested by luciferase reporter activity assays. **e** AKT inhibitor MK-2206 treatment inhibits the expression of miR-21 and miR-34a induced by Bmi-1 overexpression (*left panel*), and activated AKT increases the expression of miR-21 and miR-34a inhibited by Bmi-1 knock down (*right panel*). Fold change of miR-21 and miR-34a in gastric cancer cells was analyzed by QRT-PCR. **f** Schematic representation of the molecular mechanisms and regulation pathways in Bmi-1 regulating gastric cancer stem cell-like properties. *Error bars* in all panels represent the mean ± SD (*P < 0.05, **P < 0.01)

In the above-mentioned experiments, we found that Bmi-1 downregulates PTEN and upregulates pAKT (Fig. 8a). Since PTEN is a well-known target gene of miR-21[32], Bmi-1 may regulate PTEN via miR-21. Previous studies have also shown that Bmi-1 may inhibit the expression of PTEN by direct binding to the PTEN promoter in nasopharyngeal carcinoma cells [41]. So, we conducted ChIP test to verify whether Bmi-1 may bind to the PTEN promoter in gastric cancer cells. The results showed that Bmi-1 really binds to the PTEN promoter region, which were similar to those in previous study [41] (Fig. 8b), suggesting that Bmi-1 may directly inhibit the expression of PTEN and thereafter activate AKT.

It has been reported that AKT can activate NF-kB [42], so we suspected that Bmi-1 may regulate NF-kB and miR-21/miR-34a via activating AKT. First, we overexpressed AKT in Bmi-1 knockdown cells or control cells and found that activated AKT can increase phosphalated p65(pp65), which is activated p65 protein, enhance the aggregation of p65 in cell nucleus, and activate NF-kB transcriptional activity and can also reverse the decreased pp65 and NF-kB transcriptional activity induced by Bmi-1 knockdown (right panels of Fig. 8c, d, lower panel of Additional file 10: Figure S6); meanwhile, AKT inhibitor MK-2206 treatment can inhibit the increased pp65, aggregation of p65 in cell nucleus and NF-kB transcriptional activity induced by Bmi-1 overexpression (left panels of Fig. 8c, d, upper panel of Additional file 10: Figure S6), suggesting that Bmi-1 activates NF-kB via AKT. Further, we found that overexpression of AKT increased the expression of miR-21 and miR-34a and can also reverse the decreased expression of miR-21 and miR-34a induced by Bmi-1 knockdown; meanwhile, AKT inhibitor treatment can inhibit the increased expression of miR-21 and miR-34a induced by Bmi-1 overexpression (Fig. 8e), indicating that Bmi-1 upregulates miR-21 and miR-34a via activating AKT.

## Discussion

Over the past two decades, research on CSCs has attracted great interest and also made great progress. As an oncogene and stem cell marker, Bmi-1 can maintain the self-renewal of CSCs in some tumors, including breast cancer and glioma, but whether Bmi-1 is implicated in the regulation of gastric CSCs is still unclear. In this study, we isolated stem cell-like cells by serum-free microsphere culture and found that Bmi-1 was overexpressed in microsphere cells. Furthermore, Bmi-1 overexpression increased microsphere formation rate, anti-cancer drug resistance, cell migration, and stem cell markers expression, while Bmi-1 knockdown decreased these parameters in GC cells Clinical samples analysis showed that Bmi-1 expression in GC tissues was associated with regional lymph node metastasis and distant ovary metastasis and positively correlated with the expression of stem cell markers. These results suggested that Bmi-1 positively regulates the stem cell-like characteristics of GC cells.

Bmi-1 is a transcriptional inhibitor and the earliest research found that Bmi-1 downregulated the expression of p16 and p19 by inhibiting INK4a/ARF and thus regulates cell proliferation and senescence [29]. Douglas et al. found that Bmi-1 may promote the development of Ewing’s sarcoma through p16 independent mechanisms, and Bmi-1 knockdown induced expression changes of hundreds of downstream genes [30]. We previously found that Bmi-1 can influence breast cancer cells proliferation and tumorigenicity through regulating pAKT [31]. Song et al. found that Bmi-1 may inhibit PTEN expression, then activate PI3K/AKT/Snail pathway and therefore regulate EMT and metastasis of nasopharyngeal cancer cells [41]. These results indicated that Bmi-1 may play its role by regulating many downstream target genes. However, the mechanisms of Bmi-1 have not been fully elucidated, especially in regulating stemness.

miRNAs plays important roles in the process of cell proliferation, apoptosis, and carcinogenesis by interacting with mRNAs, IncRNAs, and other endogenous RNAs [43, 44]. Recently, some studies suggested that miRNAs were involved in regulation of CSCs. We speculated that Bmi-1, as an exogenous gene-silencing factor, might also regulate the expression of a variety of genes via miRNAs and found that miR-21, miR-34a, and miR-125a-5p were regulated by Bmi-1. Of these three miRNAs, miR-21 was most significantly affected by Bmi-1. In gastric cancer tissues, miR-21 had a significantly positive correlation with Bmi-1 expression. It was reported that miR-21 is highly expressed in many kinds of malignant tumor tissues and might act as an oncogene [45]. In breast cancer and colorectal cancer, miR-21 is involved in regulation of EMT and stemness [46, 47]. In gastric cancer, overexpression of miR-21 promotes cell growth, invasion, drug resistancem and EMT by inhibiting PTEN and P53 [48, 49]. It has also been found that miR-21 expression in gastric CSCs is higher than in parental cells [50]. In our study, in vitro experiments found that miR-21 was highly expressed in microsphere which enrich stem cell-like cells, and it may enhance the self-renewal, drug resistance, migration, and stem cell markers expression in GC cells, and clinical sample investigation found that miR-21 was highly expressed in GC tissues and positively correlated with lymph node metastasis and nerve invasion. These results suggested miR-21 positively regulates stem cell-like properties of GC cells. Further, we conducted co-transfection to simultaneously change the expression of Bmi-1 and miR-21 and found that upregulation of miR-21 restored the stem cell-like characteristics of GC cells inhibited by Bmi-1 knockdown; and downregulation of miR-21 inhibited the enhancement of stem cell-like characteristics induced by Bmi-1 overexpression. Therefore, it confirmed that miR-21 may mediate the function of Bmi-1 in regulating the stem cell-like characteristics of GC cells. Since miR-21 may regulate the expression of a variety of downstream target genes, Bmi-1 may form a complex regulatory network via miR-21. We tested two important downstream target genes of miR-21, p53 and PTEN, and did find Bmi-1 may regulate the downstream p53 and PTEN-AKT pathways via miR-21. Previous studies have shown that Bmi-1 may directly bind to PTEN promoter in nasopharyngeal carcinoma cells [41]. In this study, we also found that Bmi-1 binds to PTEN promoter in GC cells, suggesting Bmi-1 may directly inhibit the expression of PTEN. Taken together, these results suggested that in addition to the direct effect, Bmi-1 may indirectly regulate PTEN-AKT via miR-21 therefore forming bypass pathways to enhance its activating effect on AKT.

MiR-34a acts as a tumor suppressor and is downregulated in some kinds of cancer [51, 52]. It negatively regulates stem cell-like characteristics of glioma cells through downregulating c-Met and NOTCH [53] and inhibits the growth, invasion and metastasis of GC by targeting PDGFR and MET [51]. In present study, we found that miR-34a is downregulated in GC tissues and negatively correlates with aggressive tumor phenotype. Furthermore, in vitro studies showed that miR-34a inhibited cells proliferation and microsphere formation, decreased drug resistance, and migration potential. These results suggested that miR-34a acts as a tumor suppressor, and negatively regulates stem cell-like characteristics in GC cells. However, why Bmi-1 upregulates miR-34a which has opposite functions? We conceived there might be a negative feedback pathway between Bmi-1 and miR-34a and Bmi-1 inhibit its own overexpression and functions by inducing miR-34a. It was reported that c-Myc is a target gene of miR-34a [37], and c-Myc binds to t Bmi-1 promoter and upregulates its expression. Therefore, miR-34a may regulate Bmi-1 via c-myc. Our experiments showed that after miR-34a upregulation, Bmi-1 and c-Myc expression was inhibited, while c-Myc overexpression increased the inhibited expression of Bmi-1, confirming miR-34a downregulates Bmi-1 by targeting c-Myc. Further, we found that Bmi-1 overexpression restored stem cell-like characteristics inhibited by miR-34a upregulation. These results confirm our assumption that miR-34a negatively regulates stem cell-like characteristics of GC cells by negative feedback regulation of Bmi-1. The complicated negative feedback loop can also explain why we did not found the correlation between the expression of Bmi-1 and miR-34a in GC tissues. In addition, our study found a new mechanism and pathway by which miR-34 negatively regulates stemness through downregulating Bmi-1.

What is the mechanism of Bmi-1 to regulate miR-21 and miR-34a? Studies have shown that Bmi-1 regulates NF-kB activity by influencing its nuclear-plasma distribution [39, 54], and NF-kB can binds to the promoter region of miR-21 and miR-34a [40, 55] . Thus, we hypothesized that Bmi-1 may regulate miR-21 and miR-34a through activating NF-kB. In our study, reporter assay and western analysis confirmed that Bmi-1 may increase NF-kB aggregation in cell nucleus and activate its transcriptional activity. ChIP test confirmed the binding of NF-kB to miR-21 and miR-34a promoter, and Bmi-1 increases the binding. Furthermore, NF-kB inhibitors can inhibit the upregulation of miR-21 and miR-34a induced by Bmi-1 overexpression. These data confirmed Bmi-1regulates miR-21 and miR-34a expression via activating NF-kB. Further, as it was found that AKT can activate NF-kB, we suspected Bmi-1 may regulate NF-kB and miR-21/miR-34a via activating AKT. We found that AKT overexpression can really activate NF-kB and increase miR-21/miR-34a expression, while AKT inhibitor treatment can inhibit the increased NF-kB activity and upregulation of miR-21 and miR-34a induced by Bmi-1 overexpression, indicating that Bmi-1 upregulates NF-kB activity and miR-21/miR-34a expression via activating AKT.

## Conclusions

In conclusion, Bmi-1 positively regulates stem cell-like properties in GC cells via upregulating miR-21. Bmi-1 directly binds to PTEN promoter and regulates PTEN/AKT and indirectly regulates PTEN/AKT via miR-21 therefore forming bypass pathways to enhance its activating effect on AKT. MiR-34a negatively regulates stem cell-like characteristics by negative feedback regulation of Bmi-1. Bmi-1 upregulates miR-21/miR-34a expression by activating AKT-NF-kB pathway. Thus, we clarify a new mechanism and complex regulation pathway (Fig. 8f) by which Bmi-1 regulates stem cell-like characteristics of GC cells.

## Additional files

Additional file 1: Supplemental methods and materials. (DOCX 34 kb) Additional file 2: Figure S1. Tumorigenic spheres are derived from MKN45 gastric cancer cell line overexpressed stem cell markers. (DOC 258 kb) Additional file 3: Figure S2. Representative figures of Bmi-1 and several CSC-related proteins in gastric tumors, its surrounding normal tissues, and paired metastatic cancer samples. (DOC 6222 kb) Additional file 4:Table S1. The expression of Bmi-1 in paired primary and ovary metastatic tissues of gastric cancer ( McNemar test). (XLS 14 kb) Additional file 5: Table S2. The correlation between Bmi-1 and stem cell markers in gastric cancer tissues. (XLS 14 kb) Additional file 6:Table S3. Frequencies of altered expression of Bmi-1,miR-21 and miR-34a in the 74 gastric cancer tissues. (XLS 14 kb) Additional file 7: Figure S3. miR-21 inhibitor suppresses stem cell-like properties of gastric cancer cells MKN45. (DOC 867 kb) Additional file 8: Figure S4. miR-21 silencing inhibits stem cell-like characteristics of gastric cancer cells which were enhanced by Bmi-1 overexpression. (DOC 930 kb) Additional file 9: Figure S5. Inhibition of miR-34a promotes stem cell-like properties in gastric cancer cells. (DOC 756 kb) Additional file 10: Figure S6. Bmi-1 effects the aggregation of p65 in cell nucleus via AKT ﻿tested﻿ by immunofluorescence staining. (DOC 774 kb)

## Abbreviations

Bmi-1: B cell-specific Moloney murine leukemia virus integration site 1
ChIP: Chromatin immunoprecipitation
CSC: Cancer stem cell
EMT: Epithelial-mesenchymal transition
EpCAM: Epithelial cell adhesion molecule
IHC: Immunohistochemical analyses
miRNA: MicroRNA
QRT-PCR: Quantitative real time RT-PCR
SP: Side population
