# Supplementary material for: Bmi-1 regulates stem cell-like properties of gastric cancer cells via modulating miRNAs
Source: J Hematol Oncol. 2016 Sep 20;9:90. doi: 10.1186/s13045-016-0323-9 (PMC5029045; doi:10.1186/s13045-016-0323-9)
Supplement: Additional file 1: — Supplemental methods and materials. (DOCX 34 kb) [file 13045_2016_323_MOESM1_ESM.docx]

**Methods**

*Cell reagents and methods.* An immortalized human gastric mucosal epithelial cell line (GES-1) and human GC cell lines (MKN45, SGC-7901, HGC-27, NCI-N87, MKN28, MGC-803 and AGS) were obtained from the Surgical Institution of Ruijin Hospital. These cell lines were cultured in RPMI-1640 supplemented with 10% fetal bovine serum (FBS) and antibiotics. Cell proliferation was assessed by the Cell Counting Kit-8 (CCK8) assay. For plate colony formation assay, cells were plated in a six-well plate (10^3^ cells/well) and incubated at 37℃ for 14 days. After two washes in PBS, cells were stained with 0.5% crystal violet solution and images were obtained. The crystal violet was washed away with 33% acetic acid.

*Molecular Reagents and Methods: vectors construction and virus infection.* Retroviral vectors overexpressing Bmi-1, Bmi-1 short hairpin RNA (shRNA), and pSRa-mAkt expressing constitutively active (myristylated) Akt (mAkt) were obtained from Dr. Dimri (George Washington University Medical Center) and described earlier [1, 2]. The pNF-κB-luc construct was obtained from Dr. Li Jun (Sun Yat-Sen University, China). Stable cell lines expressing Bmi-1 or other genes of interest were generated by infection of the retroviral vectors expressing the particular genes as described [3].

pGIPZ-miR-21 and pGIPZ-miR-34a were generated by shanghai sunbio medical biotechnology CO., Ltd. The pri-miR-21 and pri-miR-34a sequence were amplified from normal human genomic DNA and constructed into the lentivirus expression vector pGIPZ. miR-21 and miR-34a inhibitor were obtained from RiboBio Co., Ltd. Stable cell lines overexpressing miR-21 or miR-34a were generated by infection of the lentiviral vectors expressing the particular genes as described [4].

*Spheroid Colony Formation Assay.* Spheroid Colony Formation Assay was carried out as described previously [5]. Human GC cells were seeded in wells (1000 cells per well or otherwise indicated) of ultra-low-attachment 6-well plates (Corning Life Sciences, Acton, MA, http://www.corning.com/lifesciences) supplemented plus 2ml of DMEM/F12 medium (Invitrogen) with 10 mM HEPES, human recombinant epidermal growth factor (EGF) (Invitrogen) at the concentration of 20 ng/ml, and human recombinant basic fibroblast growth factor (bFGF) (Invitrogen) at the concentration of 10 ng/ml. After 3~4 weeks, each well was examined using light microscope and spheroid colonies in 5 random fields were counted.

*Chemo-sensitivity Experiment.* Cells were inoculated into 96-well plates (5000 cells per well) in triplicate supplied with RPMI-1640 medium containing 10% FBS, along with different concentrations chemotherapy reagent epirubicin (EPI) or irinotecan and no drug as control. The number of viable cells was evaluated after 2 days cultivation using the Cell Counting Kit-8 (CCK8) (Dojindo, Rockville, MD, http://www.dojindo.com) following the manufacturer’s instructions, and the optical absorbance at wavelength 450 nm was measured for the supernatant of each well using the plate reader Multiskan EX (Thermo Fisher Scientific Inc., Waltham, MA; http://www.thermofisher.com).

*Cell migration assay.* Cell migration ability was analyzed by the Transwell chamber assay. Cells were plated in medium without serum, and medium containing 10% FBS in the lower chamber served as chemoattractant. After 36 hours of incubation, the cells that did not migrate or invade through the pores were carefully wiped out with cotton wool. Then the inserts were stained with 20% methanol and 0.2% crystal violet, imaged, and counted with an IX71 inverted microscope (Olympus).

*Immunologic reagents and Western blot.* Antibodies against CD44, CD133 were obtained from Miltenyi biotech (German), and antibody against Nanog was purchased from Santa Cruz Biotechnology (Santa Cruz, CA). Antibodies against Bmi-1, Oct4, SOX2, PTEN, total-AKT, phospho-AKT (pAKT), p65 and phospho-p65 (pp65) were obtained from Cell Signaling (Danvers, MA) and used at a 1: 1000 dilution. Anti-β-actin antibody (Santa Cruz, CA) was used at a 1:5000 dilution. The whole cell lysates were harvested using cell lysis buffer supplemented with a protease inhibitor cocktail (Sigma), and western blotting analyses were performed as described [6].The extraction of nuclear proteins and cytoplasmic proteins used the kit from WEIAO BioTech Ltd(Shanghai, China) and was performed as manufacturer’s commendation.

*Clinical samples.* 101 paraffin-embedded primary site specimens of GC and 72 ovarian metastases specimens originated from GC were obtained from the archives of the Fudan University Shanghai Cancer Center for further immunohistochemical (IHC) analysis. Another set of 74 fresh gastric tissues and paired normal mucosal tissues distant from the tumorous lesion were removed and frozen in RNA later from Sigma (St Louis, MO), and stored at -80°C until further use for QRT-PCR. The clinicopathologic variables were obtained from the medical records and the disease stages of the patients were classified according to the 2010 UICC/AJCC GC TNM staging system.

*Immunohistochemical Assay and Immunofluorescence staining.* Immunohistochemical (IHC) analyses was used to detect the expression of stem cell markers Bmi-1，Oct4，Sox2，Gli1，CD44，CD133 in samples of GC primary lesions. IHC was performed by using a highly sensitive streptavidin-biotin-peroxidase detection system as described [6]. All slides were interpreted by two independent observers in a blinded fashion. More than 10% of the cells stained with moderate or strong staining intensity were considered positive. Otherwise, the sample was considered negative.

Cellular Immunofluorescence staining was performed as described [7] and analyzed using light microscopy.

*Quantitative real time RT-PCR (QRT-PCR) assays.* Total RNA of cultured cell lines and tissue samples were extracted using TRIzol reagent (Invitrogen) according to the manufacturer’s protocol. Quantitative real-time PCR (qRT-PCR) assays were carried out to detect mRNA expression using the PrimeScript RT Reagent Kit (TaKaRa) and SYBR Premix Ex Taq (TaKaRa) according to the manufacturer’s instructions. For Bmi-1 mRNA, GAPDH acted as an internal control. The primers for QRT-PCR were: GAPDH forward (F)-5 ’GCTGAACGGGAAGCTCACTG3 ’, GAPDH reverse (R)-5’ GTGCTCAGTGTAGCCCAGGA3’; and Bmi-1 (F) 5’ TGGAGAAGGAATGGTCCACTTC 3’, Bmi-1 (R) 5’ GTGAGGAAACTGTGGATG

AGGA 3’. As for miR-21 and miR-34a, total RNA was poly (A) tailed using poly (A) polymerase and then reverse-transcribed into first-strand cDNA using miRcute miRNA cDNA kit (Tiangen). The reverse primer for QRT-PCR detecting was contained in miRcute miRNA qPCR Detection Kit (Tiangen), so we designed the forward primer for miR-21, miR-34a and 5S, an internal control. The forward primer for miRNAs: hsa-mir-21, 5’-CGGTAGCTTAGCAGACTGATGTTGA-3’; hsa-mir-34a, 5’-CGTGGCAGTGTCTTAGCTGGTTGT-3’; and 5s, 5’-GTCTACGGCCATACCACCCTGAAC-3’.Real-time PCR was conducted using 7900HT fast real-time PCR System (Applied Biosystems).

The mRNA levels of target genes in GC tissues were calculated in a relative quantification method as described [8], and when the expression showed a 2-fold increase or decrease compared with normal counterpart tissue, it was considered as an altered expression.

*In vivo tumorigenesis.* GC cells were injected subcutaneously into the flanks or abdominal cavity of SCID mice. After 10-12 weeks, mice were sacrificed by cervical dislocation. For the flanks injected mice, tumors were removed and weight of xenografts was tested. For the abdominal cavity injected mice, abdominal cavities were opened and the numbers of implantation metastasis were counted.

*Chromatin immunoprecipitation.* Chromatin immunoprecipitation (ChIP) was performed using the EZ ChIP kit (Millipore) according to the manufacturer's instructions. Briefly, chromatin from cells lysate was cross-linked with 1% formaldehyde (10 minutes at room temperature), and sheared using sonication to an average size of ∼500 bp, and then immunoprecipitated with anti-p65 (Cell signaling tech.). The ChIP-PCR primers were used to amplify a promoter region containing NF-κB putative binding sites in the miR-21 or miR-34a promoter [9, 10] and promoter region containing Bmi-1 putative binding sites in PTEN promoter [11].

*Dual fluorescence report assay.* Ten thousand cells were planted in 24-well plates and cultured for 24 h. 100 ng luciferase reporter plasmids or the control plasmid, with 10 ng of pRL-TK renilla plasmid (Promega), were transfected into GC cells using the Lipofectamin 2000 reagent (Invitrogen) according to the protocol provided by the manufacturer. After 48 h, luciferase and renilla signals were determined using the Dual Luciferase Reporter Assay Kit (Promega) according to the manufacturer’s recommendation.

**References:**

1 Guo WJ, Datta S, Band V*, et al.*. Mel-18, a polycomb group protein, regulates cell proliferation and senescence via transcriptional repression of Bmi-1 and c-Myc oncoproteins. *Mol Biol Cell* 2007;**18**:536-46.

2 Guo WJ, Zeng MS, Yadav A*, et al.*. Mel-18 acts as a tumor suppressor by repressing Bmi-1 expression and down-regulating Akt activity in breast cancer cells. *Cancer Res* 2007;**67**:5083-9.

3 Li J, Gong LY, Song LB*, et al.*. Oncoprotein Bmi-1 renders apoptotic resistance to glioma cells through activation of the IKK-nuclear factor-kappaB Pathway. *Am J Pathol* 2010;**176**:699-709.

4 Zheng B, Liang L, Wang C*, et al.*. MicroRNA-148a suppresses tumor cell invasion and metastasis by downregulating ROCK1 in gastric cancer. *Clin Cancer Res* 2011;**17**:7574-83.

5 Takaishi S, Okumura T, Tu S*, et al.*. Identification of gastric cancer stem cells using the cell surface marker CD44. *Stem Cells* 2009;**27**:1006-20.

6 Chen DL, Zeng ZL, Yang J*, et al.*. L1cam promotes tumor progression and metastasis and is an independent unfavorable prognostic factor in gastric cancer. *J Hematol Oncol* 2013;**6**:43.

7 Xu D, O TM, Shartava A*, et al.*. Isolation, characterization, and in vitro propagation of infantile hemangioma stem cells and an in vivo mouse model. *J Hematol Oncol* 2011;**4**:54.

8 Lu YW, Li J, Guo WJ. Expression and clinicopathological significance of Mel-18 and Bmi-1 mRNA in gastric carcinoma. *J Exp Clin Cancer Res* 2010;**29**:143.

9 Yang CH, Yue J, Fan M*, et al.*. IFN induces miR-21 through a signal transducer and activator of transcription 3-dependent pathway as a suppressive negative feedback on IFN-induced apoptosis. *Cancer Res* 2010;**70**:8108-16.

10 Li J, Wang K, Chen X*, et al.*. Transcriptional activation of microRNA-34a by NF-kappa B in human esophageal cancer cells. *BMC Mol Biol* 2012;**13**:4.

11 Song LB, Li J, Liao WT*, et al.*. The polycomb group protein Bmi-1 represses the tumor suppressor PTEN and induces epithelial-mesenchymal transition in human nasopharyngeal epithelial cells. *J Clin Invest* 2009;**119**:3626-36.
