# Supplementary material for: Bmi-1 regulates stem cell-like properties of gastric cancer cells via modulating miRNAs
Source: J Hematol Oncol. 2016 Sep 20;9:90. doi: 10.1186/s13045-016-0323-9 (PMC5029045; doi:10.1186/s13045-016-0323-9)
Supplement: Additional file 2: Figure S1. — Tumorigenic spheres are derived from MKN45 gastric cancer cell line overexpressed stem cell markers. (DOC 258 kb) [file 13045_2016_323_MOESM2_ESM.doc]

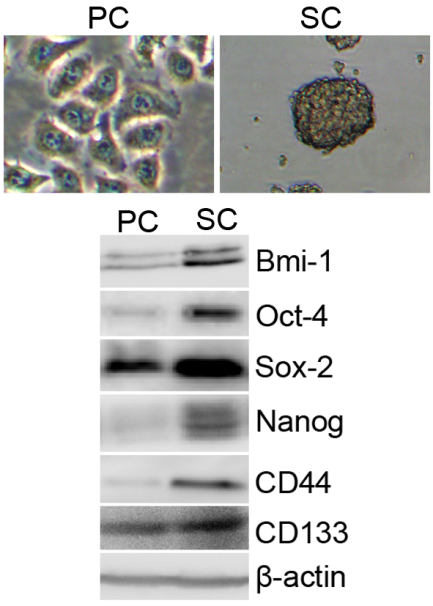
Additional file 2: Figure S1.

Figure S1.

Tumorigenic spheres are derived from MKN45 gastric cancer cell line in serum-free media containing EGF and bFGF (upper panel) and overexpressed stem cell markers including Bmi-1, Oct-4, Sox-2, Nanog, CD44, and CD133 (lower panel). The expression of stem cell markers in the cell lysis was analyzed by western blot.
