# Supplementary material for: Bmi-1 regulates stem cell-like properties of gastric cancer cells via modulating miRNAs
Source: J Hematol Oncol. 2016 Sep 20;9:90. doi: 10.1186/s13045-016-0323-9 (PMC5029045; doi:10.1186/s13045-016-0323-9)
Supplement: Additional file 3: Figure S2. — Representative figures of Bmi-1 and several CSC-related proteins in gastric tumors, its surrounding normal tissues, and paired metastatic cancer samples. (DOC 6222 kb) [file 13045_2016_323_MOESM3_ESM.doc]

Additional file 3: Figure S2.


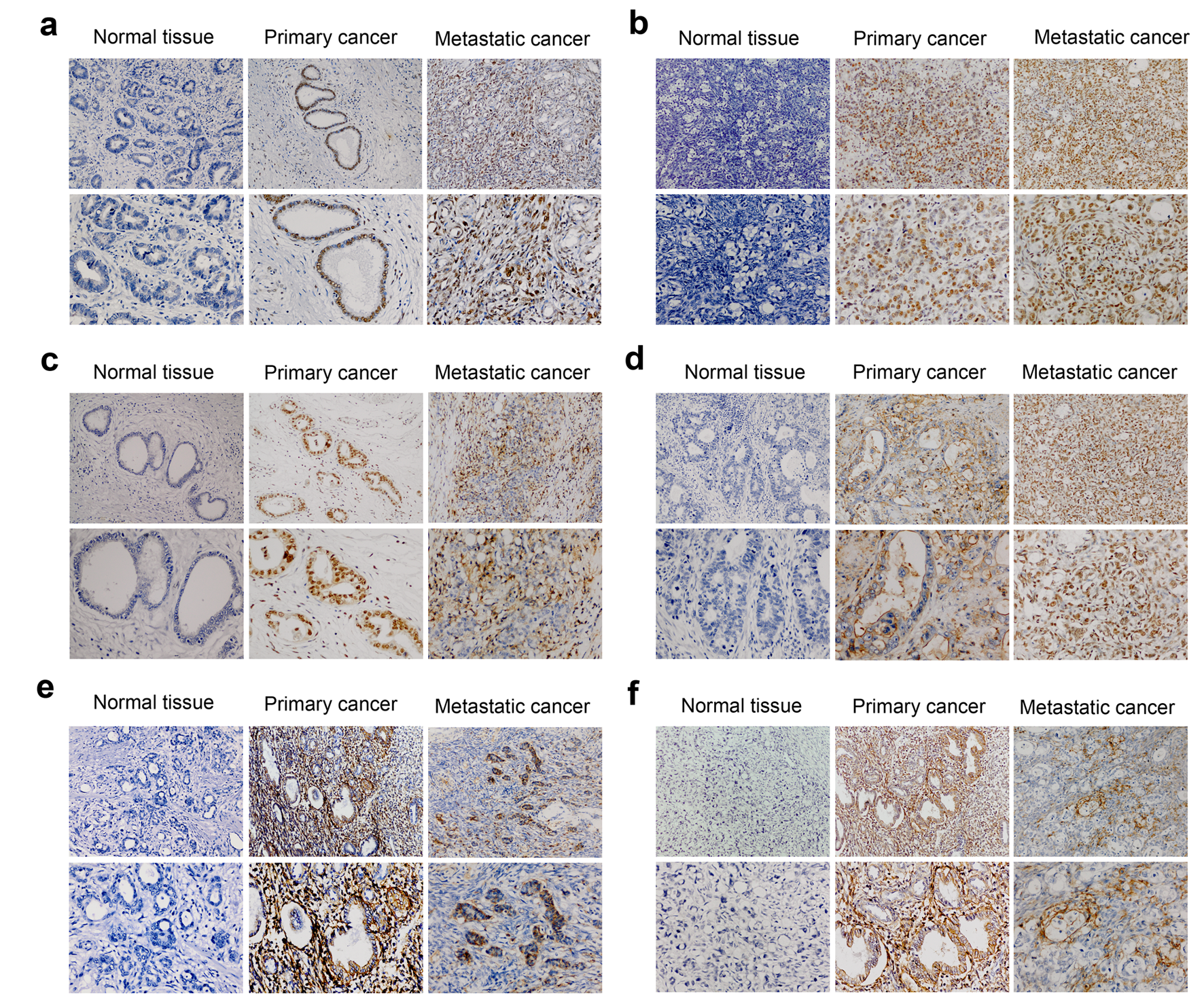


Figure S2.

Representative figures of Bmi-1 and several CSC-related proteins in gastric tumors, its surrounding normal tissues and paired metastatic cancer samples. a. primary cancer tissues and metastatic cancer tissues express more Bmi-1 compared with normal tissues. b, primary cancer tissues and metastatic cancer tissues express more Oct-4 compared with normal tissues. c. primary cancer tissues and metastatic cancer tissues expresses more Sox-2 compared with normal tissues. d. primary cancer tissues and metastatic cancer tissues express less Gli1 compared with normal tissues. e. primary cancer tissues and metastatic cancer tissues expresses more CD44 compared with normal tissues. f. primary cancer tissues and metastatic cancer tissues expresses more CD133 compared with normal tissues.
