# Supplementary material for: Bmi-1 regulates stem cell-like properties of gastric cancer cells via modulating miRNAs
Source: J Hematol Oncol. 2016 Sep 20;9:90. doi: 10.1186/s13045-016-0323-9 (PMC5029045; doi:10.1186/s13045-016-0323-9)
Supplement: Additional file 7: Figure S3. — miR-21 inhibitor suppresses stem cell-like properties of gastric cancer cells MKN45. (DOC 867 kb) [file 13045_2016_323_MOESM7_ESM.doc]

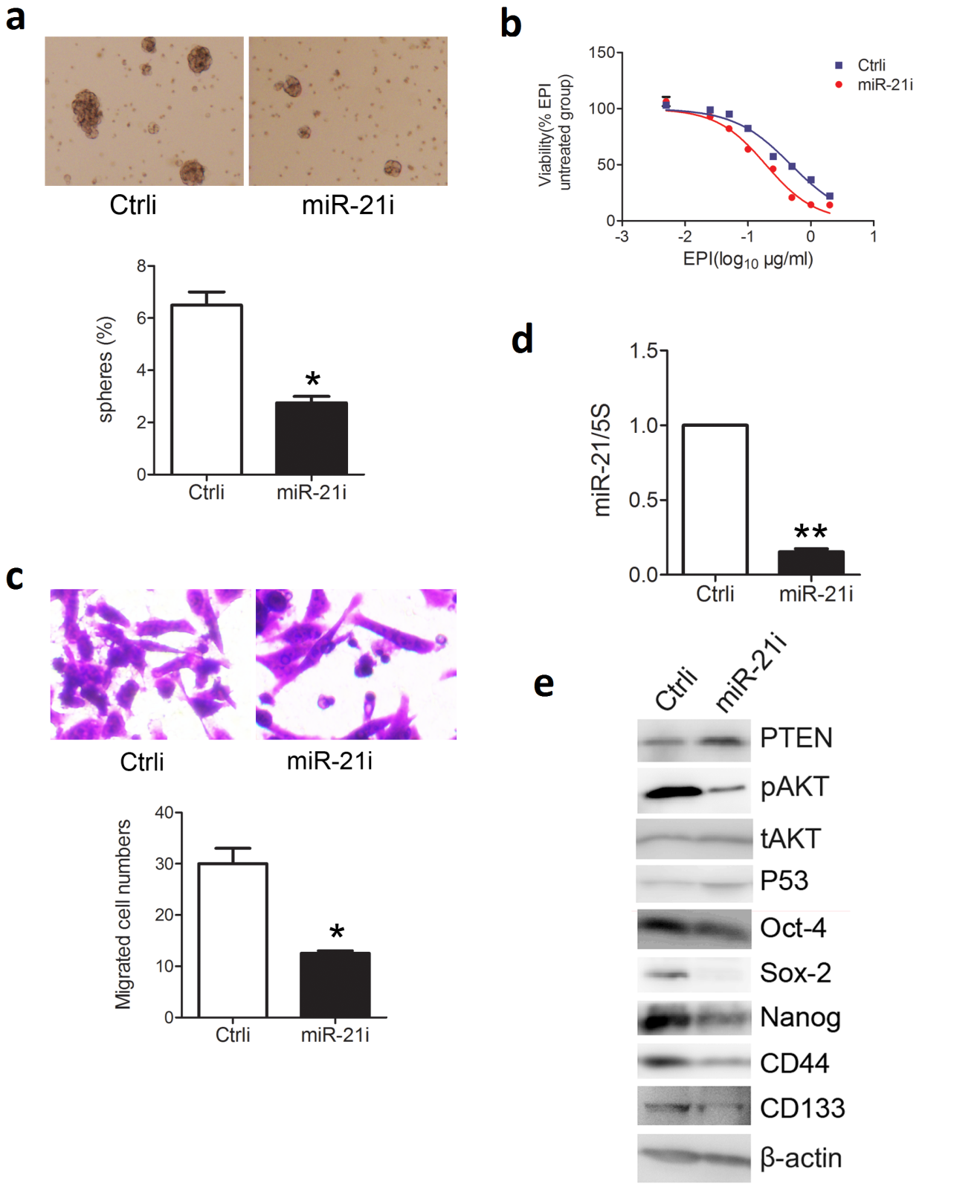
 Additional file7: Figure S3.

Figure S3. miR-21 inhibitor suppresses stem cell-like properties of gastric cancer cells. a miR-21 block inhibits microsphere formation rate in MKN45 gastric cancer cells. Microsphere formation rate was detected by serum-free culture(upper panel)and quantified (lower panel) inMKN45cells transfected with miR-21 inhibitor(miR-21i) and negative control (Ctrli). b miR-21 inhibition decreases drug resistance in MKN45 cells. Cell viability in miR-21 silencing cells (miR-21i) and control cells (Ctrli) treated with different doses of EPI for 48 hours was determinated with CCK-8. c miR-21 inhibition suppresses migration potential in MKN45 cells. Migration ability of cells was detected by Transwell Assay, and then photographed (upper pane) and quantified (lower panel). d miR-21block was confirmed in MKN45 cells after transfected with miR-21 inhibitor.Fold change of miR-21 in miR-21 silencing cells (miR-21i) and control cells (Ctrli) was analyzed by QRT-PCR. e miR-21 block suppresses the expression of stem cell markers in MKN45 cells. The expression of stem cell markers and known miR-21 target and down-stream genes (PTEN-AKT, P53) in the cell lysis was analyzed by Western blot. Error bars in all panels represent the mean ± SD (*P < 0.05, **P < 0.01as compared with control).
