# Supplementary material for: Bmi-1 regulates stem cell-like properties of gastric cancer cells via modulating miRNAs
Source: J Hematol Oncol. 2016 Sep 20;9:90. doi: 10.1186/s13045-016-0323-9 (PMC5029045; doi:10.1186/s13045-016-0323-9)
Supplement: Additional file 8: Figure S4. — miR-21 silencing inhibits stem cell-like characteristics of gastric cancer cells which were enhanced by Bmi-1 overexpression. (DOC 930 kb) [file 13045_2016_323_MOESM8_ESM.doc]

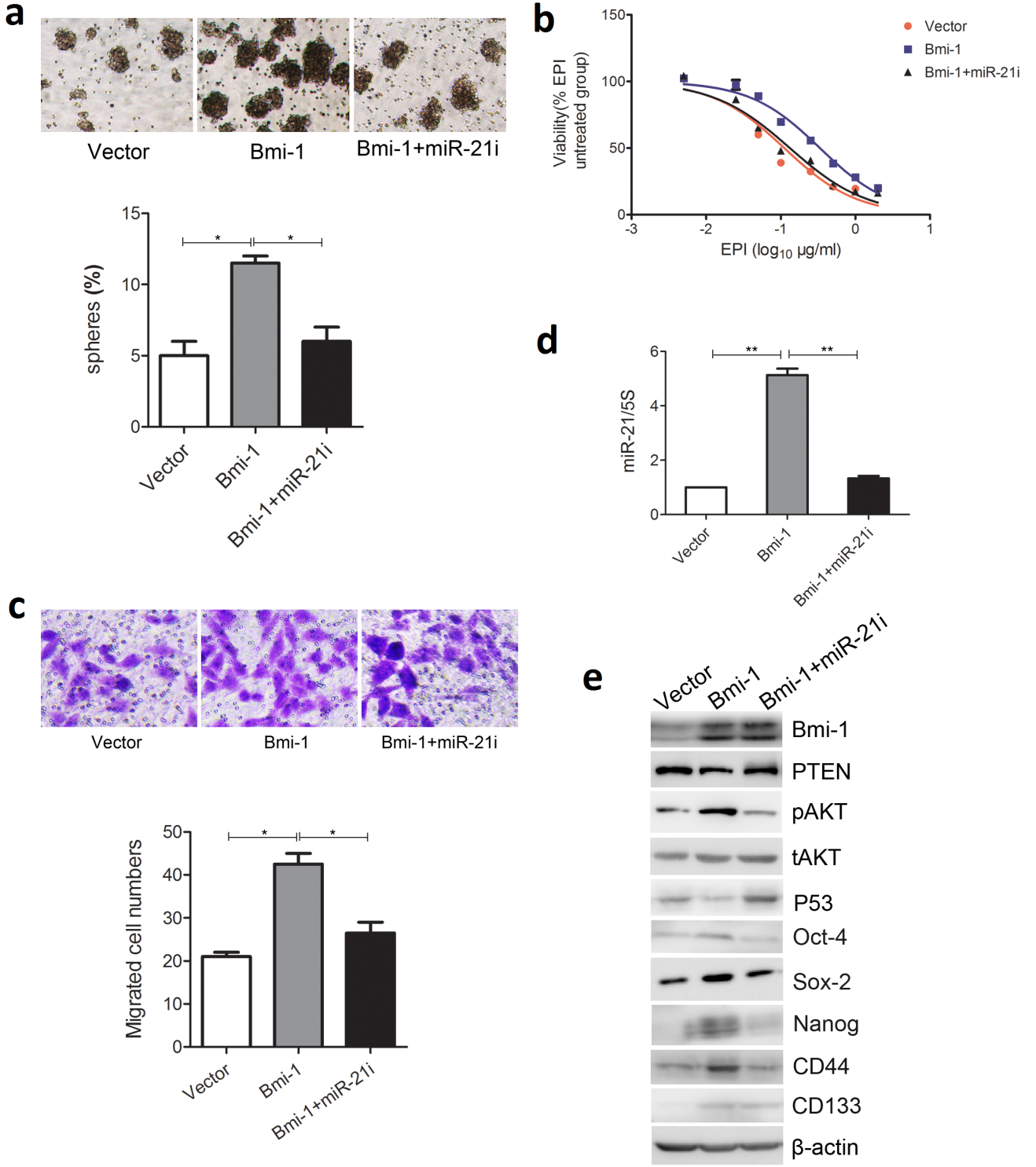
Additional file 8: Figure S4.

Figure S4. miR-21 silencing inhibits stem cells-like characteristics of gastric cancer cells which were enhanced by Bmi-1 overexpression. a miR-21 silencing inhibits microsphere formation of Bmi-1 overexpressing SGC7901 cells. Microsphere formation in control cells (Vector), Bmi-1 overexpressing cells(Bmi-1) and cells with Bmi-1overexpression and simultaneous knockdown of miR-21(Bmi-1+miR-21i)was detected by serum-free suspension culture(upper panel)and quantified (lower panel). b miR-21 block decreases drug resistance in SGC7901 cells which was enhanced by Bmi-1 overexpression. Cell viability in Vector, Bmi-1, and Bmi-1+miR-21i SGC7901 cells treated with different doses of EPI for 48 hours was determinated with CCK-8method. c miR-21 inhibition suppresses migration ability in SGC7901 cells which was induced by Bmi-1 overexpression. Migration ability in Vector, Bmi-1, and Bmi-1+miR-21i cells was tested by Transwell migration assay (upper panel) and quantified (lower panel). d miR-21 block in Bmi-1 overexpressing cells was confirmed by QRT-PCR. Fold change of miR-21 in Vector, Bmi-1, and Bmi-1+miR-21i SGC7901cellswas determinated by QRT-PCR. e miR-21silencing reverses the changes of stem cell markers and miR-21 target downstream genes (PTEN-AKT, P53)expression induced byBmi-1 overexpression. The expression of Bmi-1, stem cell markers and miR-21 target genes (PTEN-AKT, P53) in Vector, Bmi-1, and Bmi-1+miR-21i SGC7901 cells was analyzed by western blot. Error bars in all panels represent the mean ± SD (*P < 0.05, **P < 0.01).
