# Supplementary material for: Bmi-1 regulates stem cell-like properties of gastric cancer cells via modulating miRNAs
Source: J Hematol Oncol. 2016 Sep 20;9:90. doi: 10.1186/s13045-016-0323-9 (PMC5029045; doi:10.1186/s13045-016-0323-9)
Supplement: Additional file 9: Figure S5. — Inhibition of miR-34a promotes stem cell-like properties in gastric cancer cells. (DOC 756 kb) [file 13045_2016_323_MOESM9_ESM.doc]

Additional file 9: Figure S5.


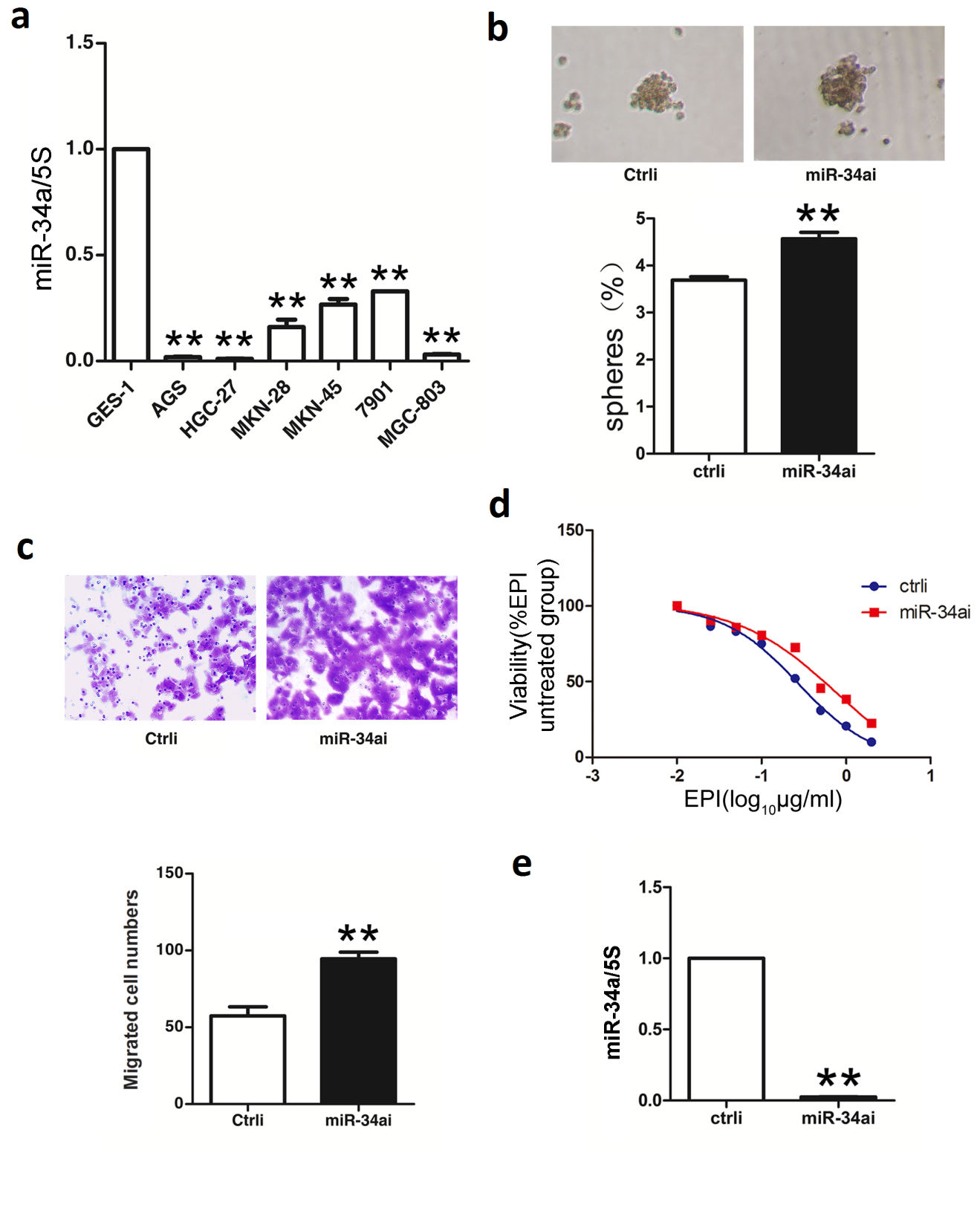
Figure S5. Inhibition of miR-34a promotes stem cell-like properties in gastric cancer cells. a Expression of miR-34a is downregulated in most gastric cancer(GC) cell lines. Expression of miR-34a in GES-1 control cells(normal immortalized human gastric mucosal epithelial cell line) and 6 human GC cell lines (AGS, HCG-27, MKN28, MKN45, SGC-7901 and MGC-803)was detected by qRT-PCR. b Silencing of miR-34a increases microsphere formation in MKN45 cells. Microsphere formation was tested by serum-free suspension culture (upper panel) and quantified (lower panel) in MKN45 cells transfected withmiR-34a inhibitor (miR-34ai) and control cells (Ctrli). c miR-34a inhibition promotes migration potential in MKN45 cells. Migration ability of cells was detected by Transwell Assay, and then photographed (upper panel) and quantified (lower panel). d miR-34a block increases drug resistance to EPI in MKN45 cells. e Inhibition of miR-34a by miRNA antagomir was confirmed by QRT-PCR. Error bars in all panels represent the mean ± SD (*P < 0.05, **P < 0.01 as compared with control).
