# Supplementary material for: Bmi-1 regulates stem cell-like properties of gastric cancer cells via modulating miRNAs
Source: J Hematol Oncol. 2016 Sep 20;9:90. doi: 10.1186/s13045-016-0323-9 (PMC5029045; doi:10.1186/s13045-016-0323-9)
Supplement: Additional file 10: Figure S6. — Bmi-1 effects the aggregation of p65 in cell nucleus via AKT ﻿tested﻿ by immunofluorescence staining. (DOC 774 kb) [file 13045_2016_323_MOESM10_ESM.doc]

Additional file 10: Figure S6.


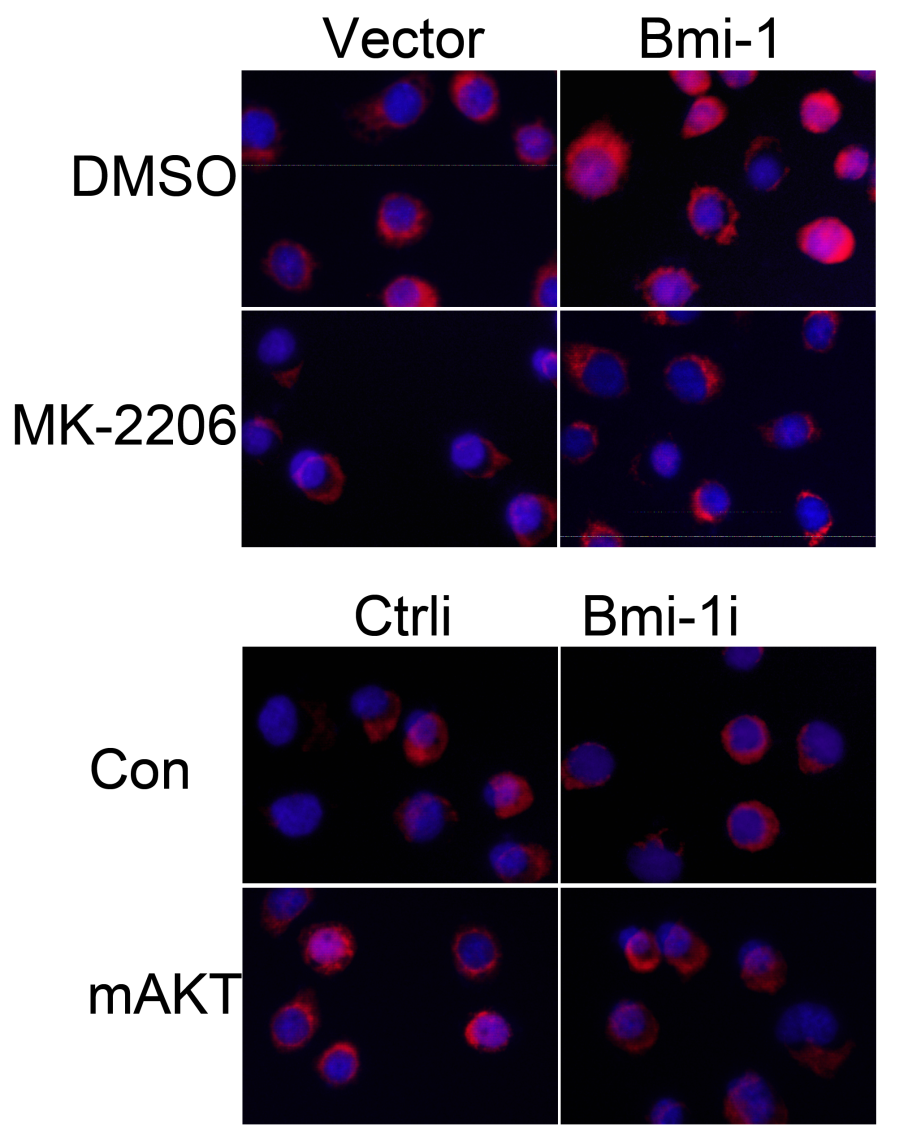
Figure S6 Activated AKT increases the aggregation of p65 in cell nucleus decreased by Bmi-1 knockdown(upper panel), and AKT inhibitor MK-2206treatmentreduces the aggregation of p65 in cell nucleus induced by Bmi-1 overexpression (lower panel).Cells were immunostained with an anti-p65 antibody and DAPI.The red signal signifies staining for p65 protein, while the blue signal represents nuclear DNA staining with DAPI.
